# Identification and characterisation of a Theileria annulata proline‐rich microtubule and SH3 domain‐interacting protein (TaMISHIP) that forms a complex with CLASP1, EB1, and CD2AP at the schizont surface

**DOI:** 10.1111/cmi.12838

**Published:** 2018-04-03

**Authors:** Sandra Huber, Tulin Karagenc, Dominic Ritler, Sven Rottenberg, Kerry Woods

**Affiliations:** ^1^ Institute for Animal Pathology, Vetsuisse Faculty University of Bern Bern Switzerland; ^2^ Department of Parasitology, Faculty of Veterinary Medicine Adnan Menderes University Aydin Turkey; ^3^ Institute of Parasitology, Vetsuisse Faculty University of Bern Bern Switzerland

**Keywords:** adaptor proteins, BioID, CD2AP, host–parasite interactions, microtubules, Theileria

## Abstract

Theileria annulata is an apicomplexan parasite that modifies the phenotype of its host cell completely, inducing uncontrolled proliferation, resistance to apoptosis, and increased invasiveness. The infected cell thus resembles a cancer cell, and changes to various host cell signalling pathways accompany transformation. Most of the molecular mechanisms leading to Theileria‐induced immortalization of leukocytes remain unknown. The parasite dissolves the surrounding host cell membrane soon after invasion and starts interacting with host proteins, ensuring its propagation by stably associating with the host cell microtubule network. By using BioID technology together with fluorescence microscopy and co‐immunoprecipitation, we identified a CLASP1/CD2AP/EB1‐containing protein complex that surrounds the schizont throughout the host cell cycle and integrates bovine adaptor proteins (CIN85, 14‐3‐3 epsilon, and ASAP1). This complex also includes the schizont membrane protein Ta‐p104 together with a novel secreted T. annulata protein (encoded by TA20980), which we term microtubule and SH3 domain‐interacting protein (TaMISHIP). TaMISHIP localises to the schizont surface and contains a functional EB1‐binding SxIP motif, as well as functional SH3 domain‐binding Px(P/A)xPR motifs that mediate its interaction with CD2AP. Upon overexpression in non‐infected bovine macrophages, TaMISHIP causes binucleation, potentially indicative of a role in cytokinesis.

## 1 INTRODUCTION

Intracellular pathogens have evolved diverse mechanisms to manipulate their host cells to their advantage and to promote their propagation within the protected environment of a host cell. *Theileria annulata*, the cause of tropical theileriosis, is an apicomplexan parasite that modulates its host cell to an impressive extent. Uniquely among eukaryotic pathogens, the infected cell acquires many phenotypes reminiscent of cancer including uncontrolled proliferation (Dobbelaere, Coquerelle, Roditi, Eichhorn, & Williams, 1988; Dobbelaere & Heussler, 2003), resistance to apoptosis (Küenzi, Schneider, & Dobbelaere, 2003), and metastasis (Adamson & Hall, 1997; Chaussepied et al., 2010; Forsyth et al., 1999). T. annulata infects bovine macrophages, monocytes, and B cells (Spooner, Innes, Glass, & Brown, 1989), and infection results in extensive changes in gene expression profiles (Kinnaird et al., 2013), while causing no changes in host DNA sequence (reviewed in Cheeseman & Weitzman, 2015). The transformed phenotype is accompanied by the alteration of multiple host cell signalling pathways, including the constitutive activation of the phosphatidylinositol 3‐kinase (PI3‐K) pathway that is required for the uncontrolled proliferation of the host cell (Baumgartner et al., 2000), c‐Jun NH_2_‐terminal kinase (JNK) that renders host cells resistant to apoptosis and contributes to their invasiveness (Lizundia et al., 2006), and JAK2‐STAT3 signalling that contributes to the survival of infected cells via c‐Myc expression (Dessauge et al., 2005). In most cases, the molecular mechanisms by which *Theileria* triggers changes in leukocyte signalling remain unknown although two strategies seem likely, namely, secretion of parasite effector molecules into the host cell or sequestration of signalling molecules at the schizont surface. Most apicomplexans develop within a membrane‐enclosed parasitophorous vacuole that the parasite remodels to allow access to nutrients and to avoid lysosomal fusion (reviewed in Clough & Frickel, 2017). It is through this physical barrier that parasites such as *Toxoplasma* and *Plasmodium* interact with and manipulate their host cells. *Theileria*, on the other hand, dissolves the surrounding host cell membrane soon after invasion—a prerequisite for survival—and thus, the schizont surface provides a structural platform for direct host–parasite interactions (Shaw, Tilney, & Musoke, 1991).

*Theileria* associates closely with host cell microtubules (MTs) throughout its entire intracellular life. The transforming schizont remains intracellular and achieves its persistence within the continually dividing host cell by interacting closely with host MTs and the mitotic apparatus, ensuring its distribution to both daughter cells (Hulliger, Wilde, Brown, & Turner, 1964; Schubert et al., 2010). Considering the importance of parasite–cytoskeleton interactions in maintaining the transformed phenotype, we recently showed that the host cell MT‐associated proteins (MAPs) EB1 (Woods et al., 2013) and CLASP1 (Huber et al., 2017) are recruited to the schizont and speculated on their possible role in recruiting and stabilising MTs on the parasite surface. We discovered that the MT‐stabilising protein CLASP1 is sequestered almost completely at the parasite surface, where it remains throughout the entire host cell cycle, and we showed that the kinetochore‐binding domain of CLASP1 is necessary and sufficient for parasite interaction.

We decided to use CLASP1 as a bait to search for schizont surface‐associated proteins with the aim of identifying novel host–parasite interactions at the parasite–leukocyte interface. To achieve this goal we used proximity‐dependent biotinylation (BioID). This proved to be a useful tool for discovering previously undescribed interactions, and here we demonstrate the localisation of the adaptor proteins CD2AP, CIN85, ASAP1 and 14‐3‐3 epsilon, and the MT binding protein Jakmip1 at the parasite surface. We also show that proteins involved in nuclear transport and constituents of the nuclear pore complex (NPCs) including RanGAP1, RanBP2, Importin B1, Nup214, and Nup160 accumulate close to defined parts of the schizont surface. We show that the schizont membrane protein Ta‐p104, which we have previously described to interact with both EB1 and CLASP1 (Huber et al., 2017; Woods et al., 2013), is a part of the CD2AP/CLASP1 complex, together with a novel secreted T. annulata protein (encoded by TA20980), which we term microtubule and SH3 domain‐interacting protein (TaMISHIP). TaMISHIP localises to the schizont surface where it interacts with bovine CD2AP via conserved SH3‐binding Px(P/A)xPR motifs. Similar to Ta‐p104, TaMISHIP is capable of tracking growing MT plus ends in an SxIP‐motif dependent manner when expressed in the cytoplasm. Upon overexpression in non‐infected bovine macrophages (BoMac), TaMISHIP induces binucleation, indicative of a role in cytokinesis. Together, these data lead us to propose a role for TaMISHIP in regulating the interaction of the host cytoskeleton with the parasite.

## 2 RESULTS

### 2.1 Use of BioID to identify proteins at the T. annulata schizont surface

**Figure 1:**
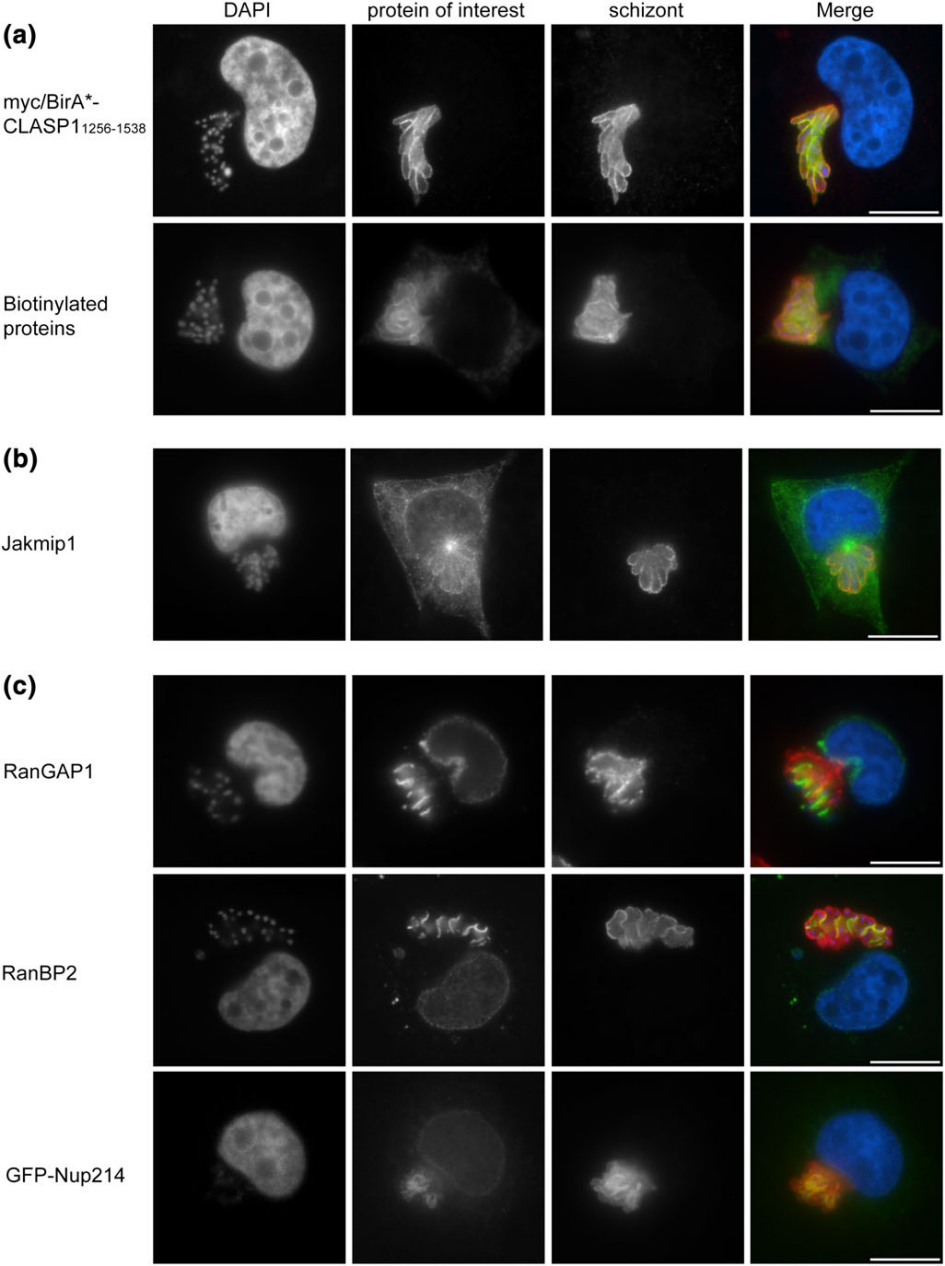
Use of BioID to identify microtubule (MT)‐binding proteins and proteins of the nuclear pore complex at the schizont surface. (a) Theileria annulata‐transformed cells (TaC12) were transduced with myc‐BirA*‐CLASP1_1256–1538_ lentivirus particles and analysed by immunofluorescence analysis. The localisation of myc‐BirA*‐CLASP1_1256–1538_ was analysed with anti‐myc labelling (green); anti‐TaSP (red) antibodies were used to label the schizont surface (top panel). TaC12_ myc‐BirA*‐CLASP1_1256–1538_ cells were incubated with 50‐μM biotin prior to fixation and analysis with FITC‐conjugated streptavidin (green); the parasite surface was labelled with anti‐p104 antibodies (red). DNA is labelled with DAPI (blue). (b) The MT‐interacting protein Jakmip1 associates with the parasite surface. TaC12 cells were stained with anti‐Jakmip1 (green), the parasite was labelled with anti‐p104 (red), and host and parasite nuclei were labelled with DAPI (blue). (c) Several proteins involved in nucleo‐cytoplasmic transport were identified with BioID and tested for proximity to the T. annulata schizont surface. TaC12 cells were stained with anti‐RanGAP1 (green, top panel), anti‐RanBP2 (green, middle panel), or transfected with GFP‐Nup214 (bottom panel). The parasite was labelled with anti‐p104 (red, top, and middle panels) or anti‐TaSP (red, bottom panel), and host and parasite nuclei were labelled with DAPI (blue). Scale bar = 10 μm

Host–parasite interactions in *Theileria*‐transformed leukocytes are poorly characterised, so we performed BioID with the aim of identifying protein–protein interaction networks that occur at the schizont surface. This approach works by fusing a promiscuous *Escherichia coli* biotin protein ligase (BirA*) to a protein of interest and expressing it in cells. Upon adding biotin to cell culture media, proteins in close proximity to the fusion protein are biotinylated and can be affinity purified using streptavidin coated beads and identified by mass spectrometry (Roux, Kim, Raida, & Burke, 2012). We fused myc‐BirA* to the kinetochore binding domain of CLASP1 (amino acids 1256–1538) that was found to be sufficient for parasite binding (Huber et al., 2017). The fusion protein was expressed in T. annulata infected cells (TaC12) by lentiviral transduction, resulting in myc‐BirA*‐CLASP1_1256–1538_ expression in approximately 60% of the population. Immunofluorescence analysis (IFA) with anti‐myc antibodies confirmed that the myc‐BirA* tag did not disrupt recruitment of CLASP1_1256–1538_ to the parasite surface (Figure 1a, top panel). Following incubation of transduced cells in culture media containing 50‐μM biotin, we used FITC‐conjugated streptavidin to confirm that proteins at and near the parasite surface were indeed biotinylated (Figure 1a, bottom panel). In nontransduced cells, no specific biotinylation of the parasite surface could be observed (not shown). Because the biotinylation reaction results in covalent biotin attachment to proximal proteins, stringent lysis conditions can be employed to solubilise protein complexes prior to affinity purification. This is an advantage when dealing with poorly soluble proteins, such as those incorporated in the parasite membrane. To test if biotinylated proteins could be detected by Western blotting following cell lysis, TaC12_myc‐BirA*‐CLASP1_1256–1538_ cells were incubated with biotin, lysed exactly as described in (Roux et al., 2012), and probed with HRP‐conjugated streptavidin. Lysates from nontransduced cells, or TaC12_myc‐BirA*‐CLASP1_1256–1538_ cells cultured without additional biotin (not shown), served as negative controls. Biotinylated proteins of approximately 80 and 130 kDa were identified in the control experiment, which likely represent endogenously biotinylated proteins such as pyruvate carboxylase (130 kDa), biotinylated subunits of β‐methyl‐crotonyl‐CoA carboxylase (80 kDa), or propionyl‐CoA carboxylase (72 kDa; Chapman‐Smith & Cronan, 1999). In TaC12_myc‐BirA*‐CLASP1_1256–1538_ cells cultured in the presence of biotin, several additional biotinylated proteins could be detected (Figure S1A). A prominent band of approximately 70 kDa corresponds to the myc‐BirA*‐CLASP1_1256–1538_ fusion protein, unsurprising seeing as BirA* is reported to biotinylate itself (Roux et al., 2012).

To facilitate mass spectrometry identification of biotinylated proteins, 2.5 × 10^8^ transduced and nontransduced TaC12 cells were incubated with 50‐μM biotin, subjected to stringent lysis, and affinity purified by using streptavidin coated beads. Proteins were digested on beads as described in (Roux et al., 2012) and analysed by mass spectrometry. By using this approach, we identified 2 *Theileria* proteins (encoded by TA08425 and TA03615) with 5 and 10 unique peptides, respectively, and with no peptides identified in the negative control (Table 1 and S1). TA08425 encodes the schizont membrane protein Ta‐p104, which we previously showed to interact with host EB1 via an SxIP motif (Woods et al., 2013). TA03615 encodes a hypothetical protein, which is predicted to be secreted and also contains an SxIP‐motif and one FAINT (“frequently associated in *Theileria*”) domain. We recently confirmed that, like Ta‐p104, TA03615 is expressed on the schizont surface and that both TA03615 and Ta‐p104 interact (directly or indirectly) with CLASP1 (Huber et al., 2017).

**Table 1:** Theileria annulata and bovine proteins identified with confidence in the myc‐BirA*‐CLASP1_1256–1538_ experiment

						Number of unique peptides	
ID	Description	Cellular location (function)	%Coverage	#PSM	Protein Mass (Da)	TaC12	TaC12_myc‐BirA*‐CLASP1_1256–1538_
Q4UCH5	T. annulata putative uncharacterized protein (TA03615)	Secreted, host cell cytoplasm/schizont membrane	19.51	17	90499.741		10
L0 L373	*T. annulata* major schizont surface protein p104 (TA08425)	Secreted, schizont plasma membrane	10.26	20	101040.604		5
E1BGU2	CLASP1	Cytoplasm (MT plus‐end tracking protein, promotes stabilisation of dynamic MTs)	5.62	49	162254.491		8
E1BQ15	CLASP2	Cytoplasm (MT plus‐end tracking protein, promotes stabilisation of dynamic MTs)	1.88	23	168182.483		1
E3W9A2	CAP‐Gly domain‐containing linker protein 1 (CLIP‐170)	Cytoplasm (MT plus‐end tracking protein)	3.92	2	160624.438		2
A6QR54	Janus kinase and microtubule‐interacting protein 1 (Jakmip1)	cytoplasm (binds to MTs and Jak family members)	3.51	2	73271.288		2
Q28046	Adseverin	Cytoplasm (Ca^2+^‐dependent actin filament severing protein)	22.38	107	80577.919		8
H7BWW0	Coronin 1B	Cytoplasm (actin filament nucleation, inhibition of actin filament depolymerization)	18.24	19	54252.78		5
P79136‐2	F‐actin‐capping protein subunit β (isoform β‐2) (CapZB)	cytoplasm (blocking exchange of subunits at barbed ends of actin filaments)	20.22	10	30628.743		3
Q5E9F5	Transgelin‐2	Cytoplasm (actin cytoskeleton)	20.1	7	22426.497		2
F1MDH3	Talin‐1	Cytoplasm (connects actin filaments with integral membrane proteins)	3.53	4	270815.461		3
P19803	Rho GDP‐dissociation inhibitor 1 (Arhgdia)	Cytoplasm (regulation of small GTPases)	45.59	8	23421.4		4
F1MM14	CD2‐associated protein (CD2AP, CMS)	Cytoplasm (actin binding, adaptor protein)	46	85	71203.755		23
F1MVN9	SH3 domain‐containing kinase‐binding protein 1 (CIN85)	Cytoplasm (adaptor protein)	5.09	8	68540.403		2
E1B7Z1	Arf‐GAP with SH3 domain, ANK repeat and PH domain‐containing protein 1 (ASAP1)	cytoplasm (GAP activity, phosphorylated by Src kinase, adaptor protein)	5.05	7	123079.556		2
G3N016	Crk‐like protein (CRKL) (predicted)	Cytoplasm (signal transduction)	12.99	4	33386.587		3
F1MFD5	Ran GTPase‐activation protein 1 (RanGAP) (predicted)	Cytoplasm, nuclear pore complex (stably interacts with RanBP2)	12.31	8	62942.186		3
F1MXS1	E3 SUMO‐protein ligase RanBP2	Interphase: nuclear pore complex (mRNA export) Mitosis: centromere (important for chromosome segregation).	38.58	208	336780.654	1	72
F1MH31	nuclear pore complex protein Nup214 isoform (predicted)	Nucleus (nuclear pore complex), cytoplasmic in mitosis	4.87	7	211599.748		5
E1BIW0	E3 ubiquitin‐protein ligase UBR1	Cytoplasm (protein ubiquitination)	2.81	6	199466.17		3

*Note*. Two T. annulata proteins (TA03615 and TA08415) were identified by mass spectrometry that were absent from the control. Seventeen bovine proteins were identified with one or more peptide hit in the myc‐BirA*‐CLASP1_1256–1538_ sample that were absent from the control. One bovine protein (FIMXS1) was identified by 72 peptides, with one peptide identified in the control. Uniprot and GeneDB were used to assign accession numbers (ID), description, and cellular localisation. The %coverage value indicates the percentage of the protein that is covered by the peptides identified by the mass spectrometry analysis. PSM is peptide spectral match, and #PSM reflects the total number of identified peptides for one particular protein. The #peptides column lists the number of different peptides found for each protein. MT = microtubule.

### 2.2 MAPs and nuclear pore complex proteins localise close to the parasite surface

In addition to the two *Theileria* proteins, we also identified 18 bovine proteins as potential CLASP1_1256–1538_ proximal proteins that were not identified (or in the case of one protein, identified with only one peptide) in the negative control (Table 1 and S1). Because proximity‐dependent labelling does not discriminate between true interaction partners and proteins that come into transient proximity with the bait, we tested the localisation of identified proteins by IFA. Among the proteins found were several MAPs, including CLASP1 itself, CLASP2, CAP‐Gly domain‐containing linker protein 1 (CLIP‐170), and Janus kinase and MT‐interacting protein 1 (Jakmip1). The presence of endogenous CLASP1 and CLASP2 validated our approach, as we have already shown that both proteins associate with the schizont surface (Huber et al., 2017). Jakmip1 interacts with both MTs and members of the Jak family (Jak1 and Tyk2; Steindler et al., 2004). Analysis of endogenous Jakmip1 in TaC12 cells revealed an association with MTs as reported, and in many cells, the outline of the parasite was labelled with Jakmip1 (Figure 1b). TaC12 cells transiently transfected with Jakmip1‐V5 confirmed this observation, with the fusion protein localising to the centrosomes and along the length of MTs, with some weak accumulation along the parasite membrane (not shown). On the other hand, expression of GFP‐CLIP‐170, a well characterised MT + TIP (plus‐end tracking protein), revealed no convincing association with the parasite surface (data not shown). CLIP‐170 interacts with the C‐terminal EEY‐motif of EB1 via its CAP‐Gly domain (Honnappa et al., 2006), with the C‐terminal (kinetochore‐binding) domain of CLASP1 (Akhmanova et al., 2001), and with tubulin dimers (Folker, Baker, & Goodson, 2005). Considering the association of EB1, CLASP1, and tubulin with the schizont surface, it is likely that CLIP‐170 comes into transient contact with the parasite and was therefore identified in our search. Several proteins involved in actin cytoskeleton regulation (Adseverin, Coronin 1B, CapZB, Transgelin‐2, Talin‐1, and Rho GDP‐Dissociation Inhibitor [Arhgdia]) were also identified in our CLASP1_1256–1538_ BioID experiment. We tested the localisation of Talin‐1 and Coronin 1B in parasitised cells and found that upon overexpression neither GFP‐Talin1 nor mCherry‐Coronin 1B exhibited any convincing parasite localisation (not shown), suggesting that their identification in the BioID pull down is likely due to their abundance in the cytoplasm rather than a specific interaction.

RanGAP1 (Ran GTPase‐activating protein 1) and RanBP2 (Ran binding protein 2, also known as Nup358) were also identified in our BioID experiment. These proteins are associated with one another throughout the cell cycle, localising to the nuclear pore complex during interphase, and to kinetochores and the mitotic spindle during mitosis where they are involved in sister chromatid segregation (Joseph, Tan, Karpova, McNally, & Dasso, 2002; Matunis, Wu, & Blobel, 1998; Werner, Flotho, & Melchior, 2012). Indirect IFA revealed that both proteins localise to TaC12 host cell nuclear membranes during interphase as reported and also accumulate close to schizont membranes in a pattern that resembles parallel lines or sheet like structures (Figure 1c, top and middle panels). GFP‐RanGAP1 was also found to localise in the vicinity of the schizont upon overexpression in TaC12 cells (not shown), confirming our observations made with the endogenous protein. The nuclear pore complex is a large structure that forms pores in the nuclear envelope, facilitating the selective transport of macromolecules between the nucleus and the cytoplasm. It is an assembly of several nuclear pore complex components (Nups), including Nup214, which is localised to the cytoplasmic face of the nuclear pore complex in interphase cells and is required for correct cell cycle progression and nuclear‐cytoplasmic transport (van Deursen, Boer, Kasper, & Grosveld, 1996). Nup214 was identified in the CLASP1_1256–1538_ BioID experiment with five unique peptides, and we found that GFP‐Nup214 localised to the nuclear pore in TaC12 cells as expected and close to the schizont surface in a similar pattern as RanGAP1 and RanBP2 (Figure 1c, bottom panel).

### 2.3 The adaptor proteins CD2AP, CIN85, and ASAP1 are detected at the schizont surface

**Figure 2:**
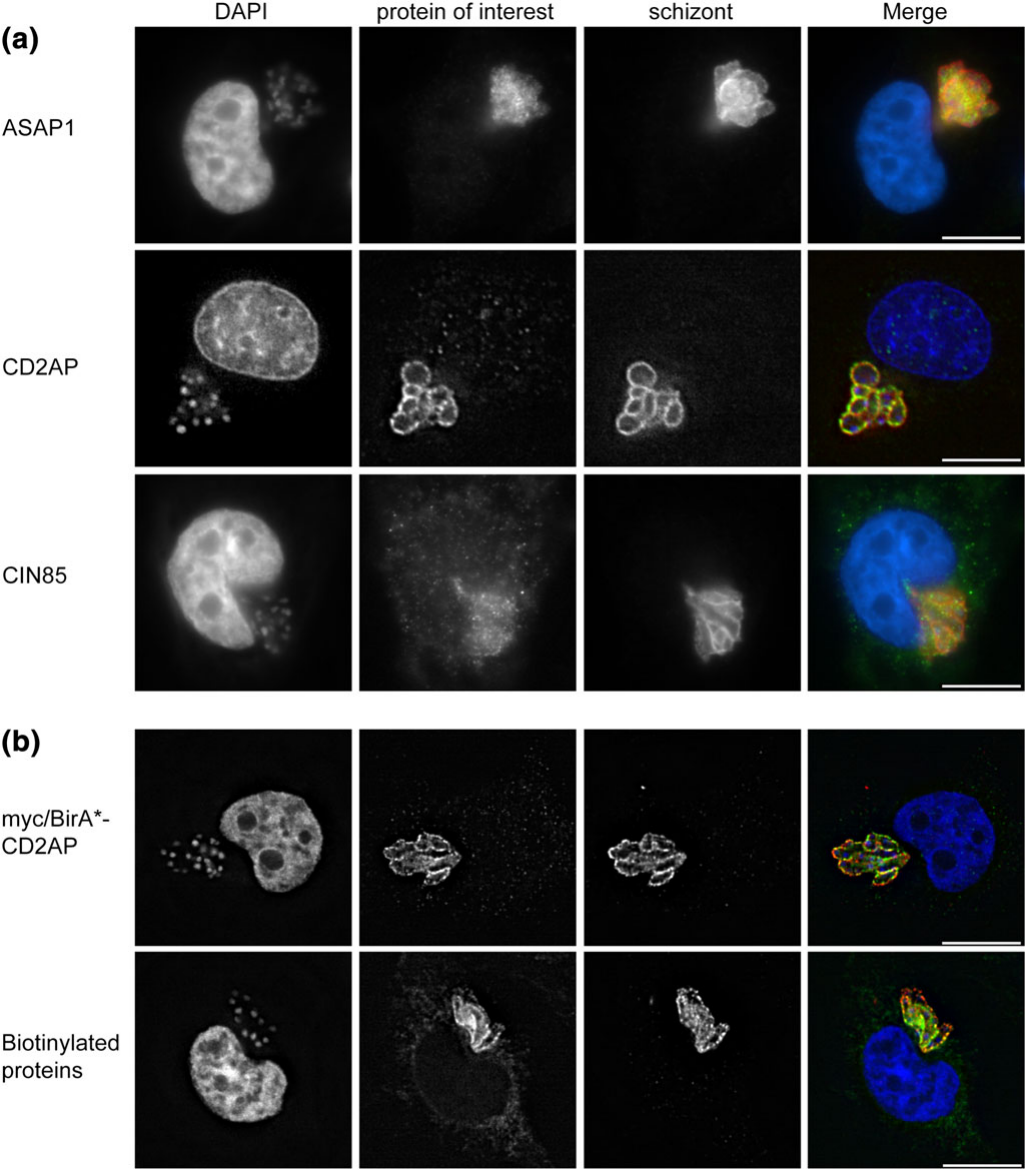
Analysis of bovine adaptor protein (ASAP1, CD2AP, and CIN85) localisation, and function of myc/BirA*‐CD2AP. (a) TaC12 cells were stained with anti‐ASAP1 (green, top panel), anti‐CD2AP (green, middle panel) or anti‐CIN85 (green, bottom panel). The parasite was labelled with anti‐p104 (red), and host and parasite nuclei were labelled with DAPI (blue). (b) TaC12 cells were transduced with the myc/BirA*‐CD2AP construct and analysed by immunofluorescence microscopy. Cells were stained with anti‐myc (green), and the parasite was labelled with anti‐TaSP (red); DNA was labelled with DAPI (blue, top panel). TaC12_myc‐BirA*‐CD2AP cells were incubated with 50‐μM biotin and subsequently stained with FITC conjugated streptavidin (green). The schizont was labelled with anti‐TaSP (red), and DNA was labelled with DAPI (blue, bottom panel). Scale bar = 10 μm

In addition to the MAPs and the nuclear pore complex proteins, a number of adaptor proteins, namely, CD2‐associated protein (CD2AP), SH3 domain‐containing kinase‐binding protein 1 (c‐Cbl‐interacting protein of 85 kDa, CIN85), Arf‐GAP with SH3 domain ANK repeat and PH domain‐containing protein 1 (ASAP1), and Crk‐like protein (CrkL), were identified. Adaptor proteins are molecules possessing two or more protein‐binding domains that function to facilitate the formation of large signalling complexes. This allows them to play pivotal roles in signal transduction, while usually possessing no catalytic activity themselves (Flynn, 2001). One prominent example is Crk (chicken tumour virus no. 10 regulator of kinase), an oncogene with the ability to transform cells. Crk consists of Src homology 2 (SH2) and SH3 protein–protein interaction domains but no catalytic domain (Mayer, Hamaguchi, & Hanafusa, 1988; reviewed in Flynn, 2001; Akagi, Murata, Shishido, & Hanafusa, 2002). Our BioID experiment identified CrkL as a possible parasite interacting protein, but GFP‐tagged CrkL and the related Crk failed to co‐localise significantly with the parasite (not shown). On the other hand, we could confirm that both endogenous ASAP1 (Figure 2a, top panel) and overexpressed tagged ASAP1 (not shown) localised close to the schizont surface. ASAP1 is known to constitutively bind to CIN85 (Kowanetz et al., 2004) and functions as a GTPase‐activating protein (GAP) for Ras‐related small GTPases (Brown et al., 1998).

The CIN85 and CD2AP/CMS family of adaptor molecules are ubiquitously expressed and have been implicated in multiple protein–protein interaction networks and signal transduction pathways (Dustin et al., 1998; Take et al., 2000; Watanabe et al., 2000; reviewed in Dikic, 2002). We investigated the localisation of CD2AP, for which 23 unique peptides were found, in *Theileria*‐infected cells. In paraformaldehyde (PFA) fixed cells, CD2AP is always found at the schizont surface (Figure 2a, middle panel). The anti‐CD2AP antibody specificity was tested in IFA with non‐infected BoMac. In BoMac cells CD2AP accumulates in the cytoplasm and the leading edge of migrating cells (not shown). This localisation of CD2AP in bovine cells is comparable with its localisation in Cos7 cells, where it is found in the cytoplasm, membrane ruffles, and the leading edges of migrating cells (Kirsch, Georgescu, Ishimaru, & Hanafusa, 1999). To exclude that CD2AP localisation in fixed cells is an artefact, and to analyse the dynamics of CD2AP localisation throughout the cell cycle, TaC12 cells were transduced with a GFP‐CD2AP construct. Live cell imaging revealed that overexpressed GFP‐CD2AP localises to the schizont surface throughout the cell cycle (Movie S1). These properties make CD2AP the second host cell protein that we identified which exclusively localises to the *Theileria* schizont surface throughout the host cell cycle. CLASP1 shows a very similar behaviour (Huber et al., 2017), whereas the interaction of other host cell proteins with the schizont varies throughout the cell cycle (e.g., PLK1, Schubert et al., 2010, and EB1, Woods et al., 2013), or depend on the presence of MTs (e.g., CLASP2, Huber et al., 2017). CIN85 shares significant structural similarity with CD2AP. Therefore, we performed the same analyses with CIN85, for which two peptides were found in the BioID experiment. Like CD2AP, endogenous CIN85 accumulates close to the schizont surface (Figure 2a, bottom panel), and its localisation to the schizont throughout the entire cell cycle was confirmed by live cell imaging of GFP‐CIN85 expressing cells (Movie S2). Although CIN85 has previously been observed in a diffuse pattern in both the cytoplasm and the nucleus in some cells (e.g., in the human prostate carcinoma [PC‐3 U] cells; Yakymovych et al., 2015), we think it is likely that the small amount of CIN85 and CD2AP observed in the nucleus of TaC12 cells is an artefact due to non‐specific antibody labelling, especially considering that ectopically expressed CD2AP/CIN85 was not found in the nucleus.

### 2.4 Use of CD2AP and CIN85 to identify further protein interaction networks at the schizont surface by using BioID

Because CD2AP and CIN85 have been implicated in multiple protein–protein interaction networks and signal transduction pathways (reviewed in Dikic, 2002), we asked the question if they could contribute to *Theileria*‐induced transformation by recruiting signalling molecules to the schizont surface. CD2AP has been shown to bind to the tumour suppressor protein p53 in the cytoplasm of human lung carcinoma cells (Panni et al., 2015), and considering that p53 has been reported to be sequestered at the surface of the *Theileria* schizont (Haller et al., 2010), we hypothesised that schizont‐associated CD2AP could play a role in recruiting p53 to the parasite. To test this, we first expressed short hairpin RNAs (shRNAs) in *Theileria*‐infected macrophages to deplete bovine CD2AP and obtained a mixed population in which the majority of cells expressed no detectable CD2AP (Figure S2A). Following selection with puromycin, CD2AP was barely detectable by Western blot (Figure S2B). Selected cells displayed a 2‐fold reduction in relative proliferation rate following 7 days in culture when compared to wild‐type (WT) TaC12 cells or those transduced with GFP‐CD2AP, or a non‐CD2AP‐targeting shRNA control plasmid (Figure S2C). To address our hypothesis that CD2AP functions to recruit signalling molecules to the parasite surface, we analysed the localisation of p53 and IKK in TaC12 cells following CD2AP depletion. Although the pattern of both p53 and IKK was rather heterogeneous in TaC12 cells (this has been previously described for IKK expression in TaC12 cells; Schmuckli‐Maurer et al., 2010), we could detect both proteins with similar patterns in close proximity to the parasite surface in both WT and CD2AP‐depleted cells (Figure S2D). These data suggested that CD2AP is unlikely to be involved in recruiting either IKK or p53 to the parasite surface. It is possible that the depletion of CD2AP achieved with shRNA expression was insufficient to abolish the recruitment of signalling molecules to the parasite; however, our attempts to use CRISPR/Cas9 to knock out CD2AP from infected macrophages failed with two guide RNA combinations. Wang et al. (2015) report that CD2AP is essential in human cancer cell lines, and we cannot exclude that CD2AP also plays an essential role in *Theileria‐*transformed cells. To further address the possibility that CD2AP and/or CIN85 are involved in forming signalling complexes at the parasite surface, we next used both adaptor proteins to target BirA* to the schizont surface and performed further BioID experiments. TaC12 cells were transduced with myc‐BirA*‐CD2AP or myc‐BirA*‐CIN85 constructs and selected with G418. The ability of the adaptor proteins to target functional myc‐BirA* fusion proteins to the schizont surface was confirmed by IFA with anti‐myc antibodies (Figure 2b, top panel, not shown for myc‐BirA*‐CIN85) and by staining with FITC‐conjugated streptavidin (Figure 2b, bottom panel, not shown for myc‐BirA*‐CIN85). In both cell lines, biotinylation mainly occurred at the schizont surface, and only a slight staining in the host cell cytoplasm could be detected. After these preliminary tests the cells were grown and prepared for lysis followed by pull down with streptavidin coated beads, as described above for CLASP1_1256–1538_. We identified 44 bovine proteins in the myc‐BirA*‐CD2AP sample with no peptides in the control sample, and 21 proteins in the myc‐BirA*‐CIN85 sample when excluding highly abundant ribosomal and histone proteins (Table S2). A number of host proteins were identified in all three BioID experiments, including CLASP1, CD2AP, CIN85, ASAP1, and RanBP2.

**Figure 3:**
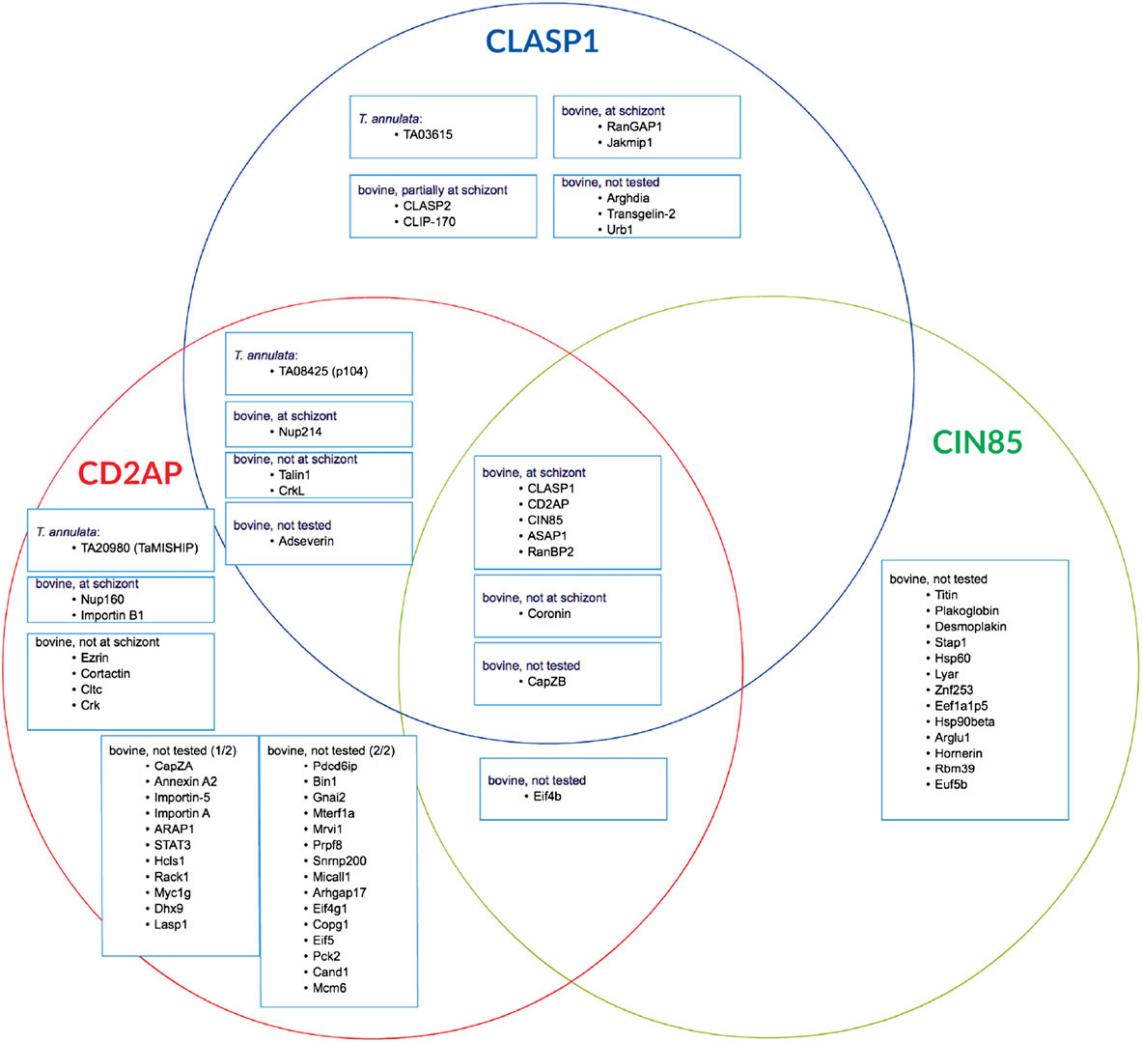
Venn diagram summarising the proteins identified in three BioID experiments. The proteins identified by mass spectrometry in three independent BioID experiments (myc‐BirA*‐CLASP1_1256–1538_, myc‐BirA*‐CD2AP, and myc‐BirA*‐CIN85) are summarised in a Venn diagram. The proteins are grouped into Theileria annulata proteins and bovine proteins that localise or do not localise to the schizont, or were not tested for schizont localisation. Immunofluorescence analysis (IFA) of proteins that were found to associate with the schizont are shown in Figures 1, 2, and S1 (Jakmip1, RanGAP1, RanBP2, GFP‐Nup214, GFP‐Nup160, GFP‐Importin B1, ASAP1, CD2AP, and CIN85). The following proteins were tested in IFA, and no association with the parasite was found: GFP‐Ezrin, GFP‐Cortactin, mCherry‐Clathrin, GFP‐Talin1, CrkL‐GFP, and Coronin1B‐pmCherry. The Venn diagram was made by using the web page https://creately.com/app/#

In addition to the three nuclear pore complex proteins identified in the CLASP1_1256–1538_ BioID experiment, the CD2AP BioID pull down identified Nup160 and three Importins (Importins A, B1, and 5)—four more proteins that are involved in transport between the nucleus and cytoplasm. Nup160 is part of the nuclear pore “Y‐complex” and is localised both to the cytoplasmic and nuclear face of NPCs where it functions as a major scaffold part of the pore (Morchoisne‐Bolhy et al., 2015). Importins bind to cargo proteins in the cytoplasm, and the complex is transported through the nuclear pore into the nucleus (reviewed in Kubitscheck & Siebrasse, 2017). Transient transfection of TaC12 cells revealed that both GFP‐Nup160 and GFP‐Importin B1 localise close to the schizont surface, as well as to the nuclear membrane or nucleus as expected (Figure S1B). The pattern of these two proteins at (or near) the schizont surface is comparable to the staining pattern of RanGAP1, RanBP2, and Nup214 (compare Figures 1c and S1B). Host and parasite proteins identified in the three BioID experiments are summarised in a Venn diagram in Figure 3.

### 2.5 CD2AP, Ta‐p104, and TaMISHIP (TA20980) interact at the T. annulata schizont surface

**Figure 4:**
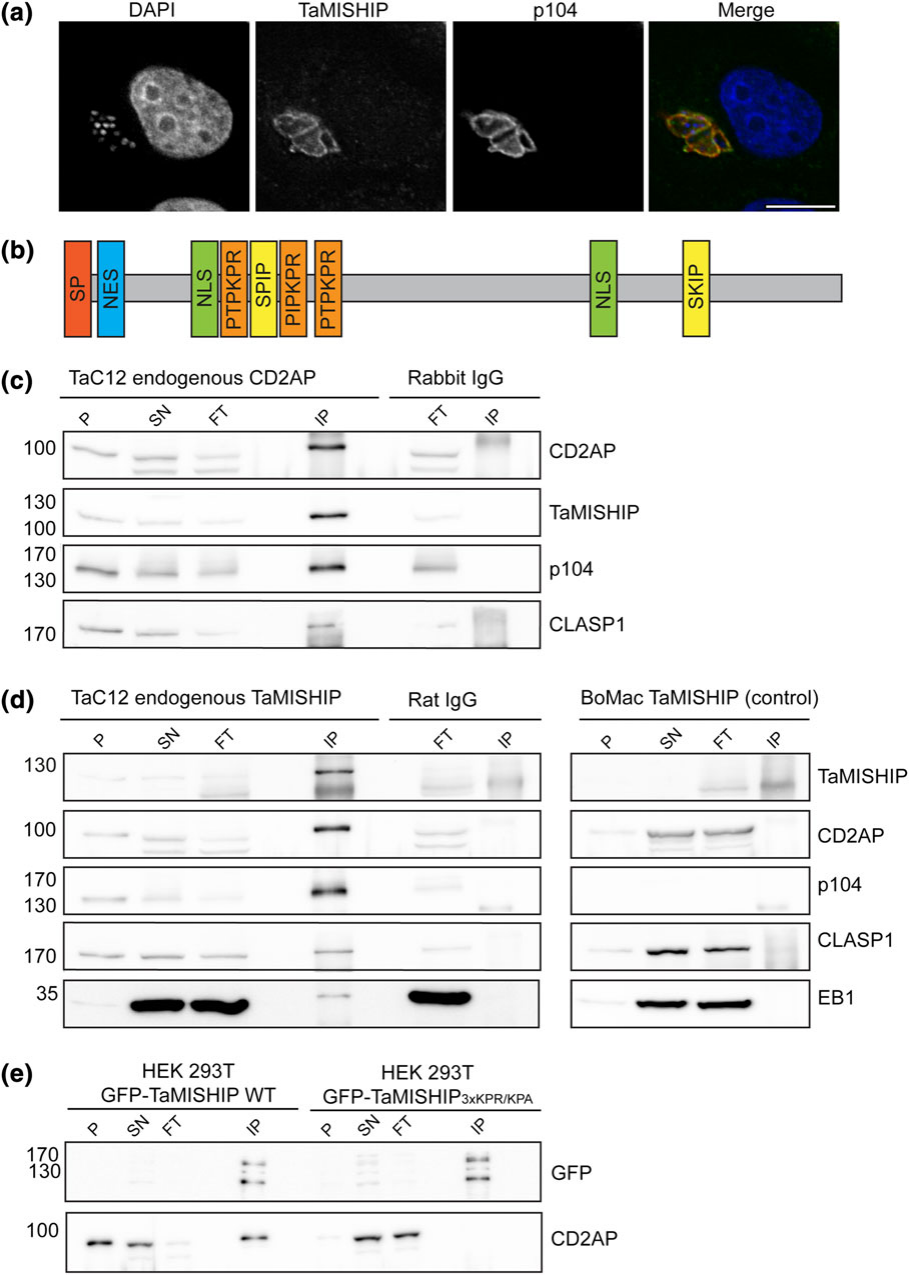
Endogenous TaMISHIP localises to the *Theileria* schizont surface and interacts with CD2AP in a protein complex on the *Theileria annulata* schizont surface. (a) Endogenous TaMISHIP was stained with anti‐TaMISHIP (green), and the parasite was labelled anti‐p104 (red). Host and parasite nuclei were labelled with DAPI (blue). Scale bar = 10 μm. (b) TaMISHIP protein structure showing different important domains of the T. annulata protein. The protein consists of 970 amino acids and comprises two EB1‐binding SxIP motifs (yellow), several domains with nuclear localisation signals (NLS, green), a domain with a nuclear export signal (NES, blue), and SH3 domain‐binding Px(P/A)xPR motifs (orange). (c) TaC12 cells were lysed and subjected to co‐immunoprecipitation (co‐IP) with rabbit polyclonal anti‐CD2AP or rabbit IgG (control) antibodies. For each sample the nonsoluble pellet (P), lysate supernatant (SN), IP flow through (FT; for each 1.5% of total amount), and 3.3% of total bound fraction after IP (IP) was analysed by SDS‐PAGE. Analysis with anti‐CD2AP antibodies confirmed the successful immunoprecipitation of CD2AP. The co‐precipitation of the parasite proteins TaMISHIP and Ta‐p104, and the host cell protein CLASP1 could be confirmed with the corresponding antibody probes. (d) TaC12 cells were lysed and subjected to co‐immunoprecipitation (co‐IP) with rat polyclonal anti‐TaMISHIP or rat IgG (control) antibodies. As a control, the anti‐TaMISHIP immunoprecipitation was also performed with non‐infected BoMac that do not express the T. annulata protein TaMISHIP. For each sample, the nonsoluble pellet (P), lysate supernatant (SN), IP flow through (FT; for each 1.5% of total amount), and 5% of total bound fraction after IP (IP) was analysed by SDS‐PAGE. Analysis with anti‐TaMISHIP antibodies confirmed the successful immunoprecipitation of TaMISHIP. The co‐precipitation of host cell CD2AP, Ta‐p104, CLASP1, and EB1 could be confirmed with the corresponding antibody probes. (e) HEK 293T cells transfected with WT GFP‐TaMISHIP or GFP‐TaMISHIP in which all three Px(P/A)xPR motifs were mutated to Px(P/A)xPA (GFP‐TaMISHIP_3XKPR➔KPA_) were lysed 16 hr after transfection and subjected to co‐IP using GFP‐TRAP magnetic beads. For each sample, the nonsoluble pellet (P), lysate supernatant (SN), IP flow through (FT; for each 1% of total amount), and 10% of total bound fraction after IP (IP) was analysed by Western blotting. Analysis with anti‐eGFP (enhanced GFP) antibodies confirmed the expression of the fusion proteins and their precipitation with GFP‐TRAP beads. GFP‐TaMISHIP is predicted to run at around 136 kDa, and the additional band detected with the anti‐GFP antibody might be degradation products of the fusion protein. Although CD2AP is co‐precipitated with WT GFP‐TaMISHIP, no interaction can be seen between mutated GFP‐TaMISHIP and CD2AP.

The BioID pull down using myc‐BirA*‐CD2AP as a bait identified two T. annulata proteins Ta‐p104 (encoded by TA08425) and one encoded by TA20980. Although we have already described some functions of Ta‐p104 (Huber et al., 2017; Woods et al., 2013), there is little information available for TA20980. TA20980 encodes a protein of 988 amino acids that contains a signal peptide of 19 amino acids with a cleavage probability of 98.2%. We propose naming the product encoded by TA20980 “TaMISHIP” (T. annulata proline‐rich microtubule and SH3 domain‐interacting protein), because we found that it can track growing MT plus ends and contains functional SH3‐binding motifs (see below). A blast search on http://blast.ncbi.nlm.nih.gov identified a homologous protein with a sequence identity of 68% in *Theileria lestoquardi* (DQ00498) and one with a sequence identity of 42% in *Theileria parva* (TP01_0380; Figure S3). Interestingly, no homologue could be found in the nontransforming T. orientalis genome, making TaMISHIP a candidate for being a transforming factor (Hayashida et al., 2012). The *T. lestoquardi* homologue was isolated as an immunogenic protein (“Clone 5”) that localises to the schizont surface and is expressed in two splice variants (Bakheit, Scholzen, Ahmed, & Seitzer, 2006). The two splice variants were also found in T. annulata and T. parva, although a function or life cycle specific expression of one or the other variant has not yet been described (Bakheit, Ahmed, & Seitzer, 2008). Microarray data shows that TA20980 is expressed in all life cycle stages, exhibiting a slight increase in gene expression in the schizont stage, with a gradual decrease through merozoites to piroplasms (Shiels and Weir, unpublished). Indirect IFA in TaC12 cells revealed that endogenous TaMISHIP is localised at the schizont surface (Figure 4a). Although TaMISHIP contains several nuclear localisation signals, and no domains that would suggest integration into the schizont membrane, we did not detect TaMISHIP in the host nucleus. We conclude that TaMISHIP is most likely secreted and then binds to other schizont surface proteins, although we cannot fully exclude the possibility that the endogenous protein is exported to the nucleus in amounts that are below the detection threshold. Analysis of TaMISHIP localisation in freshly invaded peripheral blood mononuclear cells indicates that it co‐localises with Ta‐p104 in sporozoites, likely in the microneme/rhoptry complex (Iams et al., 1990), and translocates to the surface of the parasite as it differentiates into a schizont (Figure S4A). Recently, it was reported that CD2AP is involved in *Toxoplasma gondii* invasion (Guérin et al., 2017), and so we also tested the localisation of bovine CD2AP following infection with *Theileria* sporozoites (Figure S4B). We could detect no convincing accumulation of CD2AP in the early stages of infection, suggesting that CD2AP is unlikely to play a role in invasion. We could first detect CD2AP close to the parasite surface after 24 hr when differentiation into the schizont stage had started, suggesting that CD2AP recruitment occurs only after the parasite establishes its intracellular niche.

The TaMISHIP protein sequence includes two EB1‐binding SxIP motifs, three putative SH3‐binding Px(P/A)xPR motifs, two regions consisting of several nuclear localisation signals, and one nuclear export signal (data from elm.http://eu.org; Figure 4b). Although the sequence homology between the T. annulata protein and the corresponding T. parva and *T. lestoquardi* proteins is rather low, these domains are conserved in all three species (Figure S3). Px(P/A)xPR motifs can mediate interactions with SH3 domains (Kowanetz et al., 2003), and this motif is found 3 times in TaMISHIP (Figure 4b, Figure S3). Because CD2AP and CIN85 possess three SH3 domains each, an interaction of TaMISHIP with CD2AP and/or CIN85 is possible. This was tested by immunoprecipitating (IP) endogenous CD2AP, CIN85, and TaMISHIP from *Theileria‐*infected cells. Co‐precipitated proteins were identified by mass spectrometry, and for selected proteins, the interaction was confirmed by Western blotting. CD2AP was found in TaMISHIP‐IPs, and vice versa, indicating that TaMISHIP and CD2AP interact with one another either directly or indirectly. We also detected four EB1 peptides by mass spectrometry and a faint band by Western blot following IP of TaMISHIP, indicating a weak interaction between TaMISHIP and EB1. Considering that MT plus ends come into transient contact with the parasite surface, the partial or weak interaction between these two molecules is not surprising and is similar to what we have previously described for Ta‐p104–EB1 interaction (Woods et al., 2013). Ta‐p104 and bovine CLASP1 were found repeatedly in both IPs. Together, these data suggest that Ta‐p104, CLASP1, and EB1 are part of the TaMISHIP–CD2AP complex (Figures 4c,d, S5, and S6). CIN85 was also identified by mass spectrometry in both IPs, although we could not confirm this by Western blotting. The IP of CIN85 did not, however, co‐precipitate CD2AP or TaMISHIP (Figure S7). These data might indicate that CIN85 associates rather weakly with the CD2AP/CLASP1/TaMISHIP/Ta‐p104 complex. We also found several isoforms of the adaptor protein 14‐3‐3 in both CD2AP and TaMISHIP IPs. Like CD2AP and CIN85, 14‐3‐3 family proteins can interact with multiple proteins—170 associated proteins that play roles in signal transduction or cellular communication have been identified for 14‐3‐3 gamma (Jin et al., 2004), and like CD2AP and CIN85, 14‐3‐3 epsilon is clearly recruited to the vicinity of the schizont surface (Figure S1C). The proteins identified by mass spectrometry following CD2AP, TaMISHIP, and CIN85 IPs are summarised in a Venn diagram (Figure S8), and the raw data can be found in Tables S3 and S4. Structured illumination microscopy on a DeltaVision OMX Blaze system revealed little co‐localisation of CD2AP, TaMISHIP, and Ta‐p104, although they are found to localise very close to each other at the schizont surface (Figure S9, 1 pixel = 40 nm). Another method used to confirm the close localisation of proteins is the proximity ligation assay (Duolink) that generates a fluorescent signal when two proteins are in very close proximity (less than 40 nm). This assay yields a signal for the combinations CD2AP–TaMISHIP and CD2AP–Ta‐p104, confirming the close proximity and likely interaction (direct or otherwise) of these proteins at the schizont surface. As a positive control, Duolink was performed with CLASP1 and Ta‐p104, a known interacting pair (Huber et al., 2017; Figure S10). To test whether the Px(P/A)xPR motifs found in TaMISHIP are specifically responsible for recruiting CD2AP to the schizont surface, we mutated all three motifs to Px(P/A)xPA, expressed GFP‐fusion proteins in HEK 293T cells, and subjected lysates to IP using GFP‐nanobody coated beads (GFP‐Trap). We repeatedly noticed that in the presence of WT GFP‐TaMISHIP, endogenous CD2AP was poorly soluble and was found more abundantly in the pellet (insoluble) fraction after lysis when compared to cells expressing mutated GFP‐TA20980 (Figure 4e) or control cells not expressing any fusion protein (not shown). This could indicate that TaMISHIP forms a complex with CD2AP that leads to reduced solubility. Importantly, while WT GFP‐TaMISHIP co‐precipitated CD2AP, no interaction of the mutated protein (GFP‐TaMISHIP_R173A/R189A/R205A_) with CD2AP could be detected by Western blot (Figure 4e, Figure S7D). These results confirm that the interaction between TaMISHIP and CD2AP is specific and is mediated by the Px(P/A)xPA motifs contained within TaMISHIP.

### 2.6 TaMISHIP can track MT plus ends and causes binucleation upon overexpression

**Figure 5:**
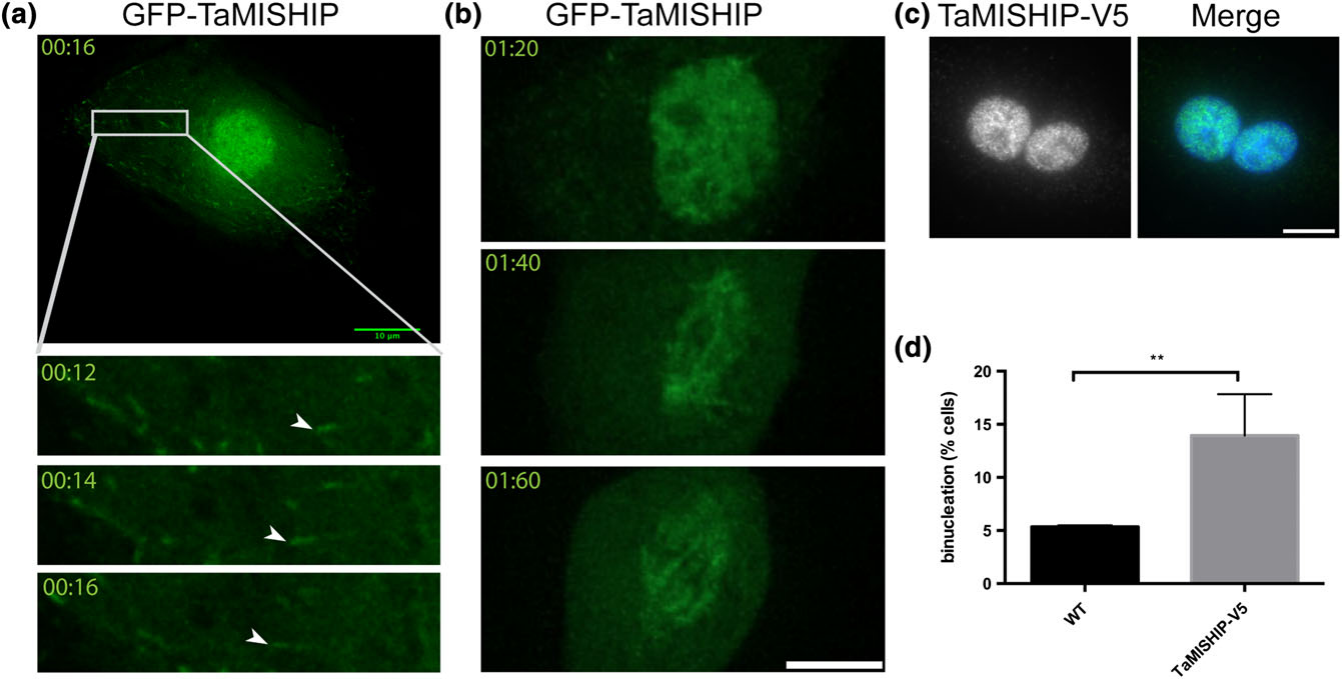
TaMISHIP localises to the nucleus, the mitotic spindle, and MT plus ends when overexpressed in non‐infected bovine macrophages. (a) Live cell imaging was performed with BoMac cells expressing GFP‐TaMISHIP, and pictures were taken every 2 s. The top panel shows a single time point from Movie S3. The region magnified in three subsequent time points (bottom panels) is indicated with a white rectangle. MT plus‐end tracking behaviour of GFP‐TaMISHIP is indicated with arrow heads. Scale bar = 5 μm. (b) Live cell imaging was performed with BoMac cells expressing GFP‐TaMISHIP, and pictures were taken every 10 min. The top panel shows a BoMac cell just prior to mitosis, when GFP‐TaMISHIP accumulates in the nucleus. The middle and bottom panels show the progression through mitosis, and the accumulation of GFP‐TaMISHIP on the mitotic spindle. The corresponding live cell imaging can be found in Movie S4. Scale bar = 5 μm. (c) TaMISHIP‐V5 was overexpressed in BoMac cells, and fixed cells were stained with anti‐V5 (green). DNA was labelled with DAPI (blue). Scale bar = 10 μm. (d) BoMac cells were transfected with TaMISHIP‐V5 and fixed 24 hr after transfection. Binucleation was counted in nontransfected and transfected cells. Shown is the mean relative number of binucleated cells in three independent experiments (*n* = 131, 90, 89; *p* = .0031)

Because TaMISHIP possesses two SxIP motifs, we considered that a localisation to growing MT plus ends is possible. We had previously tested whether the two SxIP motifs found within TaMISHIP were sufficient to mediate MT plus‐end tracking by separately expressing fragments containing one or the other of the SxIP motifs in COS7 cells, and analysing their localisation following fixation. This approach failed to reveal any interaction with MT plus ends (Woods et al., 2013). Now, to investigate the potential interaction of TaMISHIP with MT plus ends more fully, we tested the ability of full length GFP‐TaMISHIP to track MT plus ends by imaging live BoMac cells with a highly sensitive microscope. The resulting time lapse video shows that GFP‐TaMISHIP accumulates in the nucleus and the cytoplasm during interphase and that it can indeed track MT plus mends (Figure 5a, Movie S3). The discrepancy with our previous results might be caused by fixation, seeing that full‐length TaMISHIP was found to localise to MT plus ends only by live cell imaging. These observations could also indicate that the conformation of the full‐length protein is important for plus‐end tracking. Although mutation of the first SxIP motif (SPIP, aa 183–186) to SPNN did not abolish TaMISHIP localisation to MT plus ends (Movie S4), the mutation of the second motif (SKIP, aa 796–799) to SKNN completely prevented localisation of GFP‐TaMISHIP to MT plus ends (Movie S5), indicating that this motif is necessary to mediate MT plus‐end tracking. Live cell imaging as cells progressed through mitosis revealed that TaMISHIP associates with the mitotic spindle, providing further evidence of an interaction with MTs (Figure 5b, Movie S6). Finally, we observed a 2.5‐fold increase in binucleation 24 hr post transfection (Figure 5c,d, Movie S7), indicating a potential involvement of TaMISHIP in mitotic progression or cytokinetic abscission.

## 3 DISCUSSION

**Figure 6:**
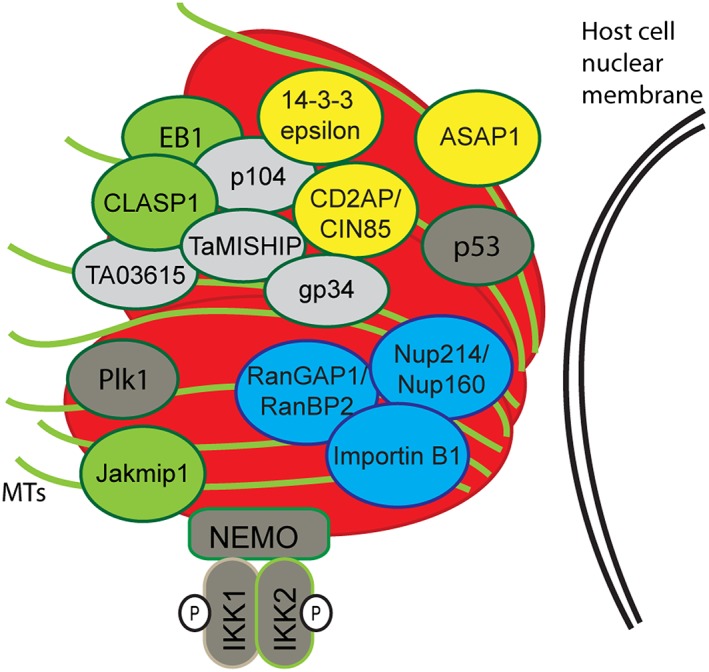
Graphical representation of host–parasite interactions on the *Theileria annulata* schizont surface. Parasite surface‐associated proteins identified in our BioID and co‐IP experiments (Ta‐p104, gp34, TaMISHIP, and TA03615) are shown in grey. Microtubule‐associating proteins found with BioID (CLASP1, Jakmip1), or previously published as binding to the parasite (EB1), are represented in green. Adaptor proteins (CD2AP, CIN85, ASAP1, and 14‐3‐3 epsilon) are represented in yellow, and proteins involved in nuclear transport (RanGAP1, RanBP2, Nup214, Nup160, and Importin B1) are represented in blue. The cell cycle‐dependent association of Plk1 with the schizont and the recruitment of the IKK signalosome complex and p53 have been described previously, and P in a circle represents phosphorylation

In this work, we have discovered a core network of interacting proteins, including CLASP1, EB1, CD2AP, Ta‐p104, and TaMISHIP (TA20980), that coats the *Theileria* surface throughout the cell cycle, and which likely plays a role in mediating interactions between the parasite and the host cytoskeleton. We also demonstrated the presence of nuclear pore complex proteins close to the surface of the schizont. This was achieved by employing BioID technology (Roux et al., 2012). BioID has been successfully used in parasitology to identify novel components of the *Trypanosoma brucei* cytoskeleton (McAllaster et al., 2015; Morriswood et al., 2013), the *Toxoplasma gondii* inner membrane complex (Chen et al., 2015), and dense granules (Nadipuram et al., 2016), as well as proteins with possible functions in gametocyte egress in *Plasmodium* (Kehrer, Frischknecht, & Mair, 2016). This is the first time that it has been applied to *Theileria*‐infected cells. We confirmed the association of 10 bovine proteins with the schizont (CD2AP, CIN85, ASAP1, 14‐3‐3 epsilon, Jakmip1, RanGAP1, RanBP2, Importin B1, Nup214, and Nup160) and show that CD2AP and CIN85 interact in a complex with CLASP1, EB1, 14‐3‐3 epsilon, and two T. annulata surface proteins (Ta‐p104 and TaMISHIP). Figure 6 summarises our current understanding of protein interactions that occur at the schizont surface.

We were particularly intrigued at the presence of several adaptor proteins at the host–parasite interface. Adaptor proteins are known to recruit signalling proteins into complexes, which led us to speculate that the schizont recruits these proteins to initiate signalling cascades on its surface. CD2AP and CIN85 share significant sequence and structural similarity and are comprised of three N‐terminal SH3 domains, a C‐terminal proline‐rich domain, multiple phosphorylatable residues, and a coiled‐coil domain that mediates homodimerisation. Further, CD2AP possesses four actin‐binding domains. Multiple binding partners have been described for CD2AP and CIN85, and they have been implicated in diverse biological processes including regulating actin cytoskeleton dynamics (Bruck et al., 2006; Hutchings, Clarkson, Chalkley, Barclay, & Brown, 2003; Kirsch et al., 1999; Zhao et al., 2013), coordinating vesicle trafficking (Cormont et al., 2003), cytokinesis (Monzo et al., 2005), and downregulating receptor tyrosine kinases (Soubeyran, Kowanetz, Szymkiewicz, Langdon, & Dikic, 2002; Szymkiewicz et al., 2002). CD2AP interacts with p85, the regulatory subunit of PI3‐K (Huber et al., 2003), and in podocytes knocked out for CD2AP, protein kinase B (PKB/Akt) is less phosphorylated and therefore less active (Schiffer, Mundel, Shaw, & Böttinger, 2004; Tossidou et al., 2007). CIN85 links B‐cell receptor signals to the canonical NF‐κB pathway. Following knock out of CIN85 in B cells, the level of both IκB and JNK1 phosphorylation was decreased, and in vitro kinase activity of the IKK complex impaired (Kometani et al., 2011). Both CD2AP and CIN85 can directly bind the tumour suppressor p53, although the functional significance of this is unknown (Panni et al., 2015). All of these signalling pathways are known to have important functions in *Theileria‐*infected cells. First, the PI3‐K pathway is constitutively active (Baumgartner et al., 2000), which leads to PKB/Akt activation and contributes to the proliferation of transformed cells (Heussler et al., 2001). Second, IKK is recruited to the schizont surface where it is constitutively phosphorylated, leading to the degradation of NF‐κB inhibitors and subsequent NF‐κB pathway activation, which is essential for the survival of *Theileria*‐infected cells (Heussler et al., 2002). Finally, p53 was found to associate with the schizont surface to prevent its translocation to the host cell nucleus, therefore inhibiting p53‐mediated apoptosis (Haller et al., 2010). Considering the contribution of these signalling pathways in *Theileria*‐induced transformation, we hypothesised that CD2AP and CIN85 could recruit signalling molecules to the schizont surface. To test this, we performed BioID experiments using CD2AP and CIN85 to target the biotin ligase to the schizont surface. Surprisingly, we did not pull down any proteins known to be involved in pro‐survival or proliferative signalling, and localisation of p53 and IKK remained unchanged in *Theileria‐*infected cells following depletion of CD2AP. This suggests that those signalling molecules present on the parasite surface (p53 and proteins of the IKK complex) are located more than 40 nm from CD2AP/CIN85 and that these adaptor proteins do not play a direct role in their recruitment.

In addition to its roles in signalling, CD2AP is involved in regulating cytokinesis. In mitosis CD2AP concentrates on midzone MTs and the midbody, and downregulation in human cells causes defects in cell separation (Monzo et al., 2005). Although depletion or overexpression of CD2AP in *Theileria‐*infected cells did not induce any detectable defects in cell cycle progression, an important finding of our work was the discovery that TaMISHIP interacts specifically with CD2AP via its Px(P/A)xPR motifs and induces binucleation when overexpressed in non‐infected cells. Interestingly, IP of both CD2AP and TaMISHIP co‐precipitated the GPI‐anchored parasite surface protein Ta‐gp34 (Figure S8, Tables S3 to S5). Ta‐gp34 has been previously characterised and, like CLASP1, CD2AP, and TaMISHIP, localises in a punctate pattern at the schizont surface (see Figure S9 for CD2AP and TaMISHIP structured illumination microscopy and Huber et al., 2017, for CLASP1). Although neither the function nor the binding partner of gp34 was elucidated, a potential involvement in cytokinesis was proposed (Xue et al., 2010). Our data suggest that Ta‐gp34 is part of a large network of (directly or indirectly) interacting proteins at the schizont surface that mediate interactions between the schizont and the host cytoskeleton and are important for regulating cytokinesis.

One specific aim of our study was to identify novel parasite molecules with the potential to interact with the host. We identified 12 T. annulata proteins in our co‐IP experiments, after excluding the highly abundant and intracellular ribosomal proteins. These proteins are summarised and described in Table S5. In order for a *Theileria* protein to qualify as a potential “host cell manipulator,” several criteria must be fulfilled. The candidate proteins should be uniquely expressed in transforming *Theileria* species, and absent from the nontransforming species T. orientalis or other nontransforming apicomplexans such as *Toxoplasma* or *Plasmodium*. The protein should be expressed in the schizont stage, as only this stage is associated with host cell transformation, and finally, the protein should be expressed at the parasite surface or secreted into the leukocyte cytosol (Shiels et al., 2006). Of the 12 proteins we identified in our experiments, only TaMISHIP fulfils these strict criteria and was chosen for further study, and indeed an attempt to use bioinformatics screening to identify potential manipulators of the host phenotype revealed TaMISHIP as a potential candidate (Hayashida et al., 2012; Shiels et al., 2006). We showed that TaMISHIP, like Ta‐p104, interacts with CLASP1 (Huber et al., 2017) and EB1 (Woods et al., 2013) and can track MT plus ends in an SxIP‐motif dependent manner. This makes it likely that TaMISHIP plays a role in the regulation of MT dynamics at the parasite surface. The overexpression of TaMISHIP in non‐infected cells led to an increase in binucleation. Considering its localisation at the mitotic spindle during mitosis, and its interaction with EB1, we propose that the most likely explanation for this is that TaMISHIP overexpression interferes with cytoskeleton dynamics and inhibits cytokinesis. On the basis of our data, we cannot exclude the possibility that the nuclear localisation of overexpressed TaMISHIP during interphase contributes to the binucleation phenotype. However, because we did not detect endogenous TaMISHIP in the nucleus of infected cells, but rather at the parasite surface, we find this rather unlikely. Taken together, our results point towards a function of the CD2AP/Ta‐p104/TaMISHIP/CLASP1/EB1 complex in mediating the interaction of the schizont with host cell MTs.

In summary, we have identified several previously unexplored host–parasite interactions in *Theileria*‐infected cells. Further work is now required to uncover other potential functions of parasite‐associated adaptor proteins and to elucidate the role that nuclear pore complex proteins play in *Theileria*‐leukocyte interactions.

## 4 EXPERIMENTAL PROCEDURES

### 4.1 Cell culture, transfections, transductions, and cell viability

*T. annulata*‐infected macrophages (TaC12) were obtained by in vitro infection of BoMac and cultured in Leibovitz 15 medium (Gibco) containing 10% fetal calf serum (FCS, BioConcept), 10‐mM Hepes pH 7.2 (Merck), 2‐mM L‐glutamine (Lonza), 70‐μM β‐mercaptoethanol (Merck), and antibiotics (Lonza) as described previously (Shiels et al., 1992). SV40‐transformed BoMac and HEK 293T cells were cultured as described previously (Schubert et al., 2010). TaC12 and BoMac cells were electroporated by using Amaxa 4D Nucleofector (Lonza) with cell solution SF and the program DS103. For GFP fusion protein expression, HEK 293T cells were transfected with FuGENE® HD Transfection Reagent (Roche) following the manufacturer's instructions. For lentiviral production, HEK 293T cells were transfected as described in (Huber et al., 2017). Media containing lentiviral particles was collected and used for transduction of TaC12 and BoMac cells. After transduction, cells expressing the gene of interest were selected by using the antibiotics G‐418 sulfate (Promega) or puromycin (Gibco). To test cell viability in cells depleted of CD2AP or overexpressing GFP‐CD2AP, 300 cells were seeded in 200‐μl Leibovitz 15 medium in 96 well plates and cultured for 3 to 7 days at 37 °C. Viability was assessed by addition of 10 μl of a 0.5‐mM solution of Resazurin (Sigma) to each well, followed by an incubation of 4 hr at 37 °C. Fluorescence intensity was measured on an EnSpire 2300 Multilabel Reader (PerkinElmer).

### 4.2 Kits and antibody production

The in situ proximity ligation assay (Duolink®) was purchased from Sigma. The GST fusion proteins (GST‐TaMISHIP_300–400_, GST‐CIN85_130–230_, and GST‐CIN85_470–620_) were expressed in BL21 Star E. coli (Invitrogen), purified by using glutathione sepharose beads (GE Healthcare), and sent to Eurogentec for antibody production and purification.

### 4.3 Primers, antibodies, and expression constructs

Primers, antibodies, and expression constructs are listed in Table S6.

### 4.4 IFA and time‐lapse imaging

Cells were seeded and grown on coverslips and fixed with 4% PFA for 10 min at room temperature. Cells were permeabilised in 0.2% Triton X‐100 and antibodies were diluted in 10% heat‐inactivated FCS in PBS. DNA was stained with DAPI (Invitrogen), and samples were mounted by using mounting media (DAKO). Fixed samples and time‐lapse experiments were analysed on a DeltaVision Elite High Resolution Microscope system (GE Healthcare) with Olympus IX‐70 inverted microscope with a CMOS camera, 60× Olympus Objective, and SoftWorx (Applied Precision) software. Samples in Figures 1, 2 (top and bottom panels), S1B,C, and S2A,D were analysed on a Nikon Eclipse 80i wide‐field microscope with a Hamamatsu Orca R2 camera using PlanApo objectives (Nikon) and the OpenLab 5 software (Improvision). Samples in Figure S9 were analysed on the DeltaVision OMX Blaze system (GE Healthcare) with sCMOS cameras at the Biozentrum in Basel, Switzerland. Fiji (ImageJ) software (Schindelin et al., 2012) and Photoshop (Adobe) were used to process the images.

### 4.5 Use of BioID to identify proteins proximal to and interacting with CLASP1_1256–1538_, CD2AP, and CIN85

The BioID approach was applied as described before (Roux et al., 2012). Briefly, TaC12 WT cells (control) and TaC12 cells expressing a myc‐BirA* fusion protein were incubated in media containing 50‐μM biotin (Serva) and subjected to stringent lysis. Biotinylated proteins were affinity purified with 600‐μl MyOne Streptavidin C1 Dynabeads (Invitrogen). Proteins were eluted from 10% of the beads for Western blot analysis, and the remaining 90% of the beads were subjected to on beads tryptic digest and analysis by mass spectrometry on an ESI‐ion trap or QTOF mass spectrometer at the Proteomics and Mass Spectrometry Core Facility, Department of Clinical Research, University of Bern, Switzerland.

### 4.6 Co‐immunoprecipitation and mass spectrometry

Wild‐type TaC12 and BoMac cells were lysed in modified RIPA buffer (50‐mM Tris–HCl pH 7.4, 150‐mM NaCl, 1‐mM EDTA, 1% NP‐40, 0.25% sodium deoxycholate, 2‐mM sodium vanadate, 25‐mM NaF, protease inhibitor cocktail [Roche]) and subjected to co‐IP. For the IP of endogenous CD2AP in TaC12 cells, 12 μg of anti‐CD2AP antibodies or 12 μg of IgG from rabbit serum (control) was bound to 1.5 mg of protein G magnetic Dynabeads (Life Technologies). The coated beads were incubated with 8 mg total protein in TaC12 cell lysates overnight at 4 °C. For the IP of endogenous CIN85 and TaMISHIP in TaC12 and BoMac (control) cells, the anti‐CIN85 and anti‐TaMISHIP anti sera (300 μl of each polyclonal affinity purified antisera) and IgG from rat serum (12 μg, control) antibodies were added to the lysates (8 mg total protein each) overnight at 4 °C. The next morning 1.5 mg of protein L magnetic Dynabeads were added for 6 hr at 4 °C. After incubation, the beads were washed 3 times with lysis buffer and 3 times with PBS. Proteins were eluted from 30% of the beads in Laemmli SDS‐sample buffer and subjected to analysis by Western blot. The remaining 70% of the beads were subjected to on beads tryptic digest and analysis by mass spectrometry on an ESI–QTOF mass spectrometer at the Proteomics and Mass Spectrometry Core Facility, Department of Clinical Research, University of Bern, Switzerland. HEK 293T cells transiently expressing GFP‐TaMISHIP fusion proteins were lysed in lysis buffer containing 0.5% NP‐40 and subjected to GFP‐TRAP co‐IP (Chromotek) following the manufacturer's instructions.

### 4.7 Sporozoite infection

A Holstein calf was infected by subcutaneous inoculation with 1 × 10^6^ cells infected with T. annulata Ankara 279. The calf was monitored for the progress of infection by taking rectal temperature and examining lymph node biopsy and blood smears for the presence of schizonts and piroplasms, respectively. Unfed nymphal *Hyalomma excavatum* ticks were applied to the ears of the calf when piroplasms were first detected in the blood smears. After the ticks detached and moulted, the sporozoite stabilate Ankara 279 was prepared as described by Brown (1987).

Peripheral blood mononuclear cells were isolated from venous bovine blood taken from a jugular vein by flotation on Ficoll‐Paque as described previously (Goddeeris & Morrison, 1988). Cells were resuspended in RPMI 1640 medium (containing 2‐mM glutamine, 5 × 10^−5^‐M 2‐mercaptoethanol, 100‐IU/ml penicillin, 100‐μg/ml streptomycin, and 10% FCS) to obtain 10^7^ cells/ml; 500 μl of cell suspension was mixed with an equal volume of T. annulata Ankara 279 stabilate diluted in culture medium to obtain an equivalent of one tick/ml. The suspension was incubated for 5 min, 30 min, 1 day, or 3 days at 37 °C with occasional agitation, then centrifuged at 200 *g* for 5 min and washed twice in PBS. The cells were fixed with 4% PFA in PBS for 10 min at room temperature, washed twice, and stored in PBS containing 0.02% sodium azide. Infections were performed at Adnan Menderes University, Turkey. Ethical statement 64583101/2013/018 from the Animal Ethics Committee of Adnan Menderes University.

## FUNDING INFORMATION

This work was supported by the Swiss National Science Foundation (SNF), project number PZ00P3_154689 awarded to Kerry Woods. The funders had no role in study design, data collection and interpretation, or the decision to submit the work for publication.

## AUTHOR CONTRIBUTIONS

S. H. and K. W. performed the conception and design of the study. S. H., K. W., T. C., and D. R. carried out the acquisition, analysis, or interpretation of the data. S. H. and K. W. did the writing of the manuscript.

## Supporting information


**Figure S1.** Analysis of myc/BirA*‐CLASP1_**1256‐1538**_ **(Western blot) and localization of Nup160, Importin B1, and 14‐3‐3 epsilon in *Theileria* infected cells. (A)** Non‐transduced TaC12 (control) and TaC12_ myc‐BirA*‐CLASP1_1256–1538_ cells were incubated in the presence of 50 μM biotin and subjected to lysis and affinity purification with streptavidin coated beads. The non‐soluble pellet (P), lysate supernatant (SN), pull down flow through (FT) (each 1% of total amount) and 10% of the total streptavidin‐bound protein (pull down, PD) were analyzed with HRP‐conjugated streptavidin. The remaining 90% of streptavidin bound sample was subjected to on‐beads tryptic digest and mass spectrometry analysis. **(B)** TaC12 cells were transfected with GFP_Nup160 (top panel) or GFP‐Importin B1 (bottom panel). The parasite was labelled with anti‐TaSP (red), and host and parasite nuclei were labelled with DAPI (blue). **(C)** TaC12 cells were stained with anti‐14‐3‐3 epsilon (green). The parasite was labelled with anti‐p104 (red), and host and parasite nuclei were labelled with DAPI (blue). Scale bar = 10 μm.
**Figure S2. Analysis of TaC12 cells depleted of CD2AP by shRNA. (A)** shRNA targeting CD2AP was expressed in TaC12 cells, and cells were labelled with anti‐CD2AP (green) prior to selection. The parasite was labelled with anti‐p104 (red), host and parasite DNA was labelled with DAPI. One cell is shown which was probably not transduced and still expresses CD2AP, while CD2AP is not detectable in neighboring cells. Scale bar = 10 μm. **(B)** After selection with puromycin, TaC12 cells expressing a CD2AP targeting shRNA (shRNA), wild type (WT) TaC12 cells, and cells expressing a non‐targeting shRNA (shRNA control) were lysed and analyzed by Western blotting. Anti‐CD2AP antibodies were used to show depletion of CD2AP (runs at around 100 kDa) in the shRNA expressing cell line, the anti‐CD2AP antibody detects unspecific bands at around 80 kDa and 50 kDa. Tubulin was used as a loading control. P is non‐solubilized pellet, S is supernatant after lysis. (C) Viability of WT TaC12 cells, puromycin‐selected TaC12 cells expressing a targeting (shRNA CD2AP) or a non‐targeting (shRNA control) shRNA, and TaC12 cells over‐expressing GFP‐CD2AP was analyzed by measuring reduction of resazurin. (D) Non‐selected cells expressing shRNA targeting CD2AP were stained with anti‐p53 (green, top panel) or anti‐IKKγ (green, bottom panel). Anti‐CD2AP (red) only labels the schizont in cells still expressing the protein, host and parasite DNA was labelled with DAPI (blue). Images were taken of a cell depleted for CD2AP next to a cell still expressing CD2AP to show similar recruitment of both IKKγ and p53 after depletion of CD2AP. Scale bar = 10 μm.
Figure S3. Sequence comparison of T. annulata TaMISHIP homologues in T. parva and *T. lestoquardi*. Protein sequences of T. parva (TP01_0380), T. annulata (TaMISHIP) and *T. lestoquardi* (DQ004498) were aligned and important motifs were highlighted (SxIP motifs in yellow, Px(P/A)xPR motifs in red, NES in blue and NLS in green).
Figure S4. TaMISHIP is expressed in T. annulata sporozoites, and host cell CD2AP localizes to the developing schizont within 24 hours after invasion of peripheral blood mononuclear cells. Peripheral blood mononuclear cells (PMBCs) were infected with T. annulata Ankara 279 sporozoites and were fixed and analyzed 5 min, 30 min and 1 to 3 days after invasion. (A) Cells were stained with anti‐TaMISHIP (green) and anti‐p104 (red) antibodies, host cell and parasite DNA was labelled with DAPI (blue). The upper panel shows a sporozoite 5 min after invasion, the middle panel shows cells fixed 30 min after invasion, and the bottom panel shows cells fixed 3 days after invasion. While TaMISHIP co‐localizes with p104 within sporozoites, it translocates to the developing schizont soon after invasion. (B) Cells were stained with anti‐CD2AP (green) and anti‐p104 (red) antibodies, host and parasite DNA was labelled with DAPI (blue). In the upper (5 min after invasion) and middle (30 min after invasion) no convincing association of CD2AP with the sporozoite can be detected. Within 24 hours after invasion (bottom panel), host cell CD2AP starts to accumulate at the developing schizont surface. Scale bar = 5 μm.
Figure S5. Co‐immunoprecipitation of endogenous CD2AP in TaC12 cells (whole membranes from Figure 4C). (A) The membrane was probed with only anti‐rabbit‐HRP to visualise the heavy and light chains of rabbit IgG used to perform the immunoprecipitation. (B) The membrane was probed for CIN85 (85 kDa). Even after contrast enhancement a co‐immunoprecipitation of CIN85 with CD2AP cannot be shown in Western blot. (C) The membrane was probed for Ta‐p104 that runs at around 150 kDa, and shows that Ta‐p104 is co‐precipitated with CD2AP (left panel). The membrane was reprobed with anti‐14‐3‐3 epsilon antibodies (middle panel). 14‐3‐3 epsilon runs at around 30 kDa, and a co‐precipitation with CD2AP cannot be shown in Western blot. A third reprobe for CD2AP (100 kDa) shows that CD2AP is precipitated. Unspecific bands at around 80 kDa and 50 kDa are also detected with this antibody (right panel). (D) The membrane was first probed for TaMISHIP (120 kDa) (left panel), and shows that TaMISHIP is co‐precipitated with CD2AP. Additional bands detected with the anti‐TaMISHIP antibody at 55 kDa, 80 kDA, 100 kDa and 170 kDa might be caused by unspecific binding or degradation / procession products of TaMISHIP. The membrane was reprobed with anti‐EB1 antibodies (middle panel) that detect EB1 at 35 kDa, and show that EB1 does not co‐precipitate with CD2AP. A third reprobe using anti‐CLASP1 antibodies (right panel) shows that CLASP1 (170 kDa) is co‐precipitated with CD2AP. P = pellet, SN = lysate supernatant, FT = IP flow through, IP = immunoprecipitation, rabbit IgG = control immunoprecipitation with rabbit IgG.
Figure S6. Co‐immunoprecipitation of endogenous TaMISHIP in TaC12 cells (whole membranes from Figure 4D). (A) The membrane was probed with only anti‐rat‐HRP (top left panel) to visualize the heavy and light chains of rat IgG used to perform the immunoprecipitation. The membrane was reprobed for TaMISHIP (120 kDa) (top right panel), and shows that TaMISHIP was immunoprecipitated. Additional bands detected with the anti‐TaMISHIP antibody at 55 kDa, 80 kDA, 100 kDa and 170 kDa might be caused by unspecific binding or degradation / procession products of TaMISHIP. A second reprobe using anti‐CLASP1 antibodies (bottom left panel) shows that CLASP1 (170 kDa) co‐immunoprecipitates with TaMISHIP. The band at 30 kDa is caused by a reprobe with anti‐14‐3‐3 gamma antibodies that is not shown here. (B) The membrane was probed with anti‐EB1 antibody, and after contrast enhancement a faint band at 35 kDa shows that EB1 partially co‐precipitated with TaMISHIP. (C) The membrane was probed for p104 (150 kDa) (top left panel), and shows that Ta‐p104 co‐precipitates with TaMISHIP. The band detected at 100 kDa corresponds to the heavy chain of IgG. A reprobe with anti‐14‐3‐3 epsilon antibodies (top right panel) did not confirm a co‐precipitation of 14‐3‐3 epsilon (30 kDa) with TaMISHIP in Western blot. A second reprobe using anti‐CD2AP antibodies (bottom left panel) confirms that CD2AP (100 kDa) is co‐precipitated with TA20980. Unspecific bands at around 80 kDa and 50 kDa are also detected with this antibody. (D) The membrane was probed for CIN85 (85 kDa). CIN85 co‐precipitation with TA20980 cannot be shown in Western blot. P = pellet, SN = lysate supernatant, FT = IP flow through, IP = immunoprecipitation, rabbit IgG = control immunoprecipitation with rabbit IgG, BoMac TA20980 = immunoprecipitation using anti‐TaMISHIP antibodies on non‐infected bovine macrophages.
Figure S7. Co‐immunoprecipitation of endogenous CIN85 in TaC12 cells. (A) TaC12 cell were lysed and subjected to co‐immunoprecipitation (co‐IP) with rat polyclonal anti‐CIN85 or rat IgG (control) antibodies. For each sample the non‐soluble pellet, lysate supernatant, IP flow through (1.5% of total amount each), and 5% of total bound fraction after IP was analyzed by SDS‐PAGE. Analysis with rat anti‐CIN85 antibodies confirmed the successful immunoprecipitation of CIN85. Host cell CD2AP and T. annulata TaMISHIP and Ta‐p104 did not co‐precipitate with CIN85. (B) Western blot (whole membranes) with CIN85 co‐IP samples. The membrane was first probed with only anti‐rat‐HRP antibodies (left panel) to visualize the heavy and light chains of IgG used to perform the immunoprecipitation. The same membrane was reprobed with anti‐CIN85 (middle panel, presented also in Figure S7A), and shows that CIN85 was immunoprecipitated and runs at about 85 kDa. A second reprobe using anti‐p104 antibodies (right panel) detects Ta‐p104 at around 150 kDa that does not co‐precipitate with CIN85. (C) Western blot (whole membranes) with CIN85 co‐IP samples. The membrane was first probed with anti‐TaMISHIP antibodies (left panel). TaMISHIP is predicted to run at about 120 kDa. Additional bands detected with the anti‐TaMISHIP antibody at 55 kDa, 80 kDA, 100 kDa and 170 kDa might be caused by unspecific binding or degradation / procession products of TaMISHIP. The membrane was reprobed with anti‐CD2AP antibodies (right panel) that detects CD2AP running at 100 kDa. Both TaMISHIP and CD2AP do not co‐precipitate with CIN85. P = pellet, SN = lysate supernatant, FT = IP flow through, IP = immunoprecipitation, rat IgG = control immunoprecipitation with rat IgG. (D) Western blot (whole membranes) corresponding to Figure 4E. GFP‐TaMISHIP is predicted to run at around 136 kDa, and the additional bands detected with the anti‐GFP antibody might be degradation products of the fusion protein.
Figure S8. Venn diagram summarizing the proteins identified in three co‐immunoprecipitations. The proteins identified by mass spectrometry in three independent co‐IP experiments with anti‐CD2AP, anti‐CIN85 and anti‐TaMISHIP antibodies are summarized in a Venn diagram. The proteins are grouped into T. annulata and bovine proteins. Antibody labelling of 14‐3‐3 epsilon revealed association with the schizont surface (Figure S1C). The following proteins were tested in IFA and no association with the parasite was found: myc‐IGF2BP3, G3BP2‐myc, Caprin1‐myc, 14‐3‐3 gamma (by antibody labelling). The Venn diagram was made by using the web page https://creately.com/app/#.
Figure S9. Interaction analysis of CD2AP, TaMISHIP and Ta‐p104 with structured illumination microscopy. Fixed cells were analysed by super resolution microscopy by using the DeltaVision OMX Blaze microscope. Z‐stack images were taken at 125 nm intervals, one z‐stack is shown for each protein combination. (A) TaC12 cells were stained with anti‐CD2AP (green) and anti‐p104 (red), (B) anti‐CD2AP (green) and anti‐TaMISHIP (red), and (C) anti‐TaMISHIP (green) and anti‐p104 (red) (bottom panel). Host and parasite nuclei were labelled with DAPI (blue). In each panel, a region showing parasite membranes was magnified (indicated with a white rectangle). Scale bar = 5 μm.
FigS10. Interaction analysis of CD2AP, TaMISHIP and Ta‐p104 with proximity ligation (Duolink® Sigma). In situ proximity ligation assays (PLA) were performed using different antibody combinations to assess the interaction of proteins at the schizont surface. A signal is obtained only when the two proteins of interest are within a maximal distance of 40 nm. (A) PLA with anti‐CD2AP and anti‐p104 as primary antibodies in TaC12 cells, and (B) anti‐CD2AP and anti‐TaMISHIP as primary antibodies. Unfortunately, there is no anti‐rat PLA®‐probe available, but the anti‐mouse PLA®‐probe is known to cross‐react with rat antibodies and was therefore used to detect rat anti‐TaMISHIP interactions. (C) Positive control: PLA with anti‐CLASP1 and anti‐p104 as primary antibodies in TaC12 cells. (D) Negative control: PLA with anti‐CD2AP and anti‐p104 (upper panel) and anti‐CD2AP and anti‐TaMISHIP (bottom panel) as primary antibodies in BoMac cells. (E) Negative control: PLA with only one primary antibody in TaC12 cells (upper panel CD2AP, middle panel p104, bottom panel TaMISHIP). Host and parasite nuclei were labelled with DAPI (blue). Scale bar = 10 μm.
Movie S1. Over‐expressed GFP‐CD2AP localizes to the T. annulata schizont surface during the entire cell cycle. GFP‐CD2AP localizes to the T. annulata schizont surface throughout the host cell cycle. Images were captured every 10 minutes for 15 hours by using a DeltaVision Elite widefield microscope with deconvolution.
Movie S2. Over‐expressed GFP‐CIN85 localizes to the T. annulata schizont surface during the entire cell cycle. GFP‐CIN85 localizes to the T. annulata schizont surface throughout the host cell cycle. Images were captured every 10 minutes for 15 hours by using a DeltaVision Elite widefield microscope with deconvolution.
Movie S3. Over‐expressed GFP‐TaMISHIP localizes to the nucleus and MT plus‐ends during interphase in uninfected bovine macrophages. GFP‐TaMISHIP localizes to MT plus‐ends in bovine macrophages. Images were captured every 2 seconds for 30 seconds by using a DeltaVision Elite widefield microscope with deconvolution.
Movie S4. The SPIP motif (aa 183‐186) of TaMISHIP is not required for MT plus end tracking in uninfected bovine macrophages. The SPIP motif within TaMISHIP (aa 183–186) was mutated by PCR to SPNN, and the full length protein was over‐expressed in bovine macrophages. Images were captured every 2 seconds for 30 seconds by using a DeltaVision Elite widefield microscope with deconvolution.
Movie S5. The SKIP motif (aa 796‐799) of TaMISHIP is required for MT plus end tracking in uninfected bovine macrophages. The SKIP motif within TaMISHIP (aa 796‐799) was mutated by PCR to SPNN, and the full length protein was over‐expressed in bovine macrophages. This was sufficient to abolish localization of GFP‐TaMISHIP to MT plus‐ends in bovine macrophages. Images were captured every 2 seconds for 30 seconds by using a DeltaVision Elite widefield microscope with deconvolution.
Movie S6. Over‐expressed GFP‐TaMISHIP localizes to the nucleus and mitotic spindle midzone in uninfected bovine macrophages. GFP‐TaMISHIP localizes to the mitotic spindle midzone in bovine macrophages undergoing mitosis and cytokinesis. Images were captured every 10 minutes for 6.5 hours by using a DeltaVision Elite widefield microscope with deconvolution.
Movie S7. Over‐expressed GFP‐TaMISHIP leads to binucleation in uninfected bovine macrophages. GFP‐TaMISHIP over‐expression can lead to binucleation in bovine macrophage. Images were captured every 10 minutes for 9.8 hours by using a DeltaVision Elite widefield microscope.
Table S1. T. annulata and bovine proteins identified in the myc‐BirA*‐CLASP1_1256–1538_ experiment (raw data). Mass spectrometry of the myc‐BirA*‐CLASP1_1256–1538_ sample identified six T. annulata protein that were absent from the control (WT TaC12 cells). Four of those proteins (TA13315, TA13460, TA08665 and TA05445) are predicted to be abundant cytoplasmic schizont proteins and were not pursued further. Only 18 bovine proteins were absent from the control sample, and were considered for further analysis.
Table S2. Proteins identified in the myc‐BirA*‐CD2AP and myc‐BirA*‐CIN85 experiment (raw data). Mass spectrometry of the myc‐BirA*‐CD2AP sample identified two T. annulata proteins (Ta‐p104 and TaMISHIP), and 44 bovine proteins with high confidence, and after exclusion of highly abundant proteins like ribosomal and histone proteins. In the myc‐BirA*‐CIN85 experiment no *Theileria* proteins, but 21 bovine proteins were identified with high confidence. The myc‐BirA*‐RanGAP1 experiment is not discussed in this paper.
Table S3. Proteins identified in co‐immunoprecipitations of TaC12 endogenous CD2AP (raw data). Endogenous CD2AP was precipitated from TaC12 cells and co‐precipitating proteins were identified by mass spectrometry. Mass spectrometry identified 6 T. annulata proteins and 25 bovine proteins that potentially co‐precipitated with CD2AP. Highly abundant proteins like ribosomal and histone proteins were excluded. CD2AP was identified with 43 peptides in the CD2AP‐IP sample, and with 2 peptides rabbit IgG control‐IP sample, but still considered as a relevant hit.
Table S4. Proteins identified in co‐immunoprecipitations of TaC12 endogenous TaMISHIP and CIN85 (raw data). Endogenous CIN85 and TaMISHIP were precipitated from TaC12 cells and co‐precipitating proteins were identified by mass spectrometry. Proteins that co‐precipitated with rat IgG and the IP done with the anti‐TaMISHIP antibody in non‐infected bovine macrophages (BoMac) correspond to the two control samples. In the CIN85 co‐IP 2 *Theileria* and 20 bovine proteins were identified, in the TaMISHIP co‐IP 11 *Theileria* and 53 bovine proteins were identified. Highly abundant proteins such as ribosomal and histone proteins were excluded.
Table S5. T. annulata proteins identified with confidence in co‐IPs of CD2AP, CIN85 or TaMISHIP. List of the 12 T. annulata proteins that were co‐precipitated with CD2AP, CIN85 and/or TaMISHIP, highly abundant ribosomal proteins were excluded from this list. The table summarises the expression pattern in *Theileria* life cycle (expression), protein sequences and domains (SP = signal peptide, TMD = transmembrane domain, GPI = glycosylphosphatidylinositol anchor, PEST = amino acid sequence that targets protein for rapid degradation, NLS = nuclear localization signal, FAINT = Frequently Associated in *T* *heileria* domain), and lists gene identifiers of homologues in *Theileria parva*, *Theileria orientalis* and *Toxoplasma gondii*.
Table S6. List of primers, antibodies and expression constructs.
