# Supplementary material for: Identification and characterisation of a Theileria annulata proline‐rich microtubule and SH3 domain‐interacting protein (TaMISHIP) that forms a complex with CLASP1, EB1, and CD2AP at the schizont surface
Source: Cell Microbiol. 2018 Apr 3;20(7):e12838. doi: 10.1111/cmi.12838 (PMC6033098; doi:10.1111/cmi.12838)
Supplement: Supplementary file 1 — Figure S1. Analysis of myc/BirA*‐CLASP1 1256‐1538 (Western blot) and localization of Nup160, Importin B1, and 14‐3‐3 epsilon in Theileria infected cells. (A) Non‐transduced TaC12 (control) and TaC12_ myc‐BirA*‐CLASP11256–1538 cells were incubated in the presence of 50 μM biotin and subjected to lysis and affinity purification with streptavidin coated beads. The non‐soluble pellet (P), lysate supernatant (SN), pull down flow through (FT) (each 1% of total amount) and 10% of the total streptavidin‐bound protein (pull down, PD) were analyzed with HRP‐conjugated streptavidin. The remaining 90% of streptavidin bound sample was subjected to on‐beads tryptic digest and mass spectrometry analysis. (B) TaC12 cells were transfected with GFP_Nup160 (top panel) or GFP‐Importin B1 (bottom panel). The parasite was labelled with anti‐TaSP (red), and host and parasite nuclei were labelled with DAPI (blue). (C) TaC12 cells were stained with anti‐14‐3‐3 epsilon (green). The parasite was labelled with anti‐p104 (red), and host and parasite nuclei were labelled with DAPI (blue). Scale bar = 10 μm. Figure S2. Analysis of TaC12 cells depleted of CD2AP by shRNA. (A) shRNA targeting CD2AP was expressed in TaC12 cells, and cells were labelled with anti‐CD2AP (green) prior to selection. The parasite was labelled with anti‐p104 (red), host and parasite DNA was labelled with DAPI. One cell is shown which was probably not transduced and still expresses CD2AP, while CD2AP is not detectable in neighboring cells. Scale bar = 10 μm. (B) After selection with puromycin, TaC12 cells expressing a CD2AP targeting shRNA (shRNA), wild type (WT) TaC12 cells, and cells expressing a non‐targeting shRNA (shRNA control) were lysed and analyzed by Western blotting. Anti‐CD2AP antibodies were used to show depletion of CD2AP (runs at around 100 kDa) in the shRNA expressing cell line, the anti‐CD2AP antibody detects unspecific bands at around 80 kDa and 50 kDa. Tubulin was used as a loading control. P is non‐solub [file CMI-20-na-s001.zip › CMI_12838-Sup-0003-FigS3.pdf]

```
T. parva|TP01_0380          MRLLLLSIVILSFLKISNASDKNPAKDPDKSPDKKNVDPLHHSTLHFRDVERLFATESTG
T. annulata|TA20980         MRLLLLSIVLLSILKISHASDKDPQKD----PQKKDVDPLHHVNVHLRLVQRLFSRDSTQ
T. lestoquardi|DQ004498     MRLLLLSIVLLSFLKISHASDKDPQKD----PPKKSVDPLHRVNVHLRLVHSLFSRESTH
                            *********:**:****:****:* **     * **.*****: .:*:* *. **: :**

T. parva|TP01_0380          EGDQTGPEPPEEAIGETEEEDYEEGEENEALVRSLLEDDTKTAEKIKSDQMKALDLSVLF
T. annulata|TA20980         LPDQPQP----EPIGEPEYEEYEEESDDEELARSILEENEERSKEIQKCKIQSLDFSEQD
T. lestoquardi|DQ004498     LVDQPMP----VPIGEPEQEEYEEESEDEELARSILEDDEEKAKEIQKCKIQSLDFSEQD
                              **  *      *** * *:*** .::* *.**:**:: : :::*:. ::::**:*

T. parva|TP01_0380          ELNSIIEEVESETDGEPEEESHGDTDEPDSKKEGEGAEEKKKRKKDDDSDS-DGEEGGKK
T. annulata|TA20980         LVMMVIEEMESKSDDEGEEGSDEGHHEPDQQEKEGKKEETKKRKKVDDTDDEEQKETEKK
T. lestoquardi|DQ004498     LVMSIMEEVESKSDDGGEEESEEGHVEPDQQEKVEKKEERKKRKKVGDSDDEDGKESDPS
                             :  ::**:**::*.  ** *. .  ***.:::    ** ***** .*:*. : :*   .

T. parva|TP01_0380          KRFKPKSTTDSDGKPKPESKGPPVPKPRTPKSDGDGKPKTDPKKPPVPKPRTPKSDGDGK
T. annulata|TA20980         KK--PKHQDKPEGVP-----QRPTPKPR-------TKPDTKPKPSPIPKPRTKPV-----
T. lestoquardi|DQ004498     KK--SKHQDEPEKKS-----QRPIPKPR-------SKPDKDKGQSPIPKPRTKPV-----
                            *:    *    . :          * ****       **...     *:*****

T. parva|TP01_0380          PKTETKGPPVPKPRTPKSDGDGKPKPETKGPPVPKPRTPKSKTEPKPAQQPQPQPQPQPE
T. annulata|TA20980         ----TIEKPSPTPKPRTKPGTME--------------------TPSPKAVPP--PKPKRT
T. lestoquardi|DQ004498     ----GKGKPVPKPATPQKP-------------------------------------KRT
                                    * *.*    .                                    :

T. parva|TP01_0380          QAPVPEPKKPEDKDSKGPEPMDTEPPK-----EPEDKDTKEPEDKDTKETEDKDTKEPEP
T. annulata|TA20980         KKPLAQTAP-VGSEEK----------------EDKKVEDTEPKPQEPI-----TETKPEP
T. lestoquardi|DQ004498     KTPVISSGT-VEPKETEPEPEKHDSQEDKDTKETEEPKDTEPEPEKPKETEDKEETVPEP
                            : *: .        ...                *  :. . .**: :.          . ***

T. parva|TP01_0380          MDTEPSKEPEKPKEPDGKDPDDHPEEGKKPEPG-----EDKSDDTKGKDSPKEQEKPKEP
T. annulata|TA20980         EPQQPQPITEPITEPQPKEP--EPEEPKEQSMPE-----------SGPEGPKETEDKD--
T. lestoquardi|DQ004498     VPEEP----------KETEP--EPEEPKETEPEDEKHDSQDDEDTKETEGPKDKKDEKEP
                               :*           . .:*  .*** *: .                .  :.**: :. .

T. parva|TP01_0380          EPVPQPEPTPAPQPTPVPQPEPTPAPQPTPVPQPDPTPAPEKPEDKDTKEDKKSDESKPM
T. annulata|TA20980         ------------ATGPEE--------------PKQTEPEHEKPKEQDDTEPKP-------
T. lestoquardi|DQ004498     ETVPEPETPISPGIAPEP--------------QPQPEEQPEKPKDQDKEETVPEPE----
                                           *                  :     ***:::*   *

T. parva|TP01_0380          DQQDTKDPGKQEADPQPKDGEKKDETPTDQEDSGKKGKQESDPVKPMETDPKPEPQPQPE
T. annulata|TA20980         ------------------DTEHEHEKHDSQEDKDTKERQEQED---SDKKDDSVPEPEPE
T. lestoquardi|DQ004498     ----H---------EKPKDTEPEPEKHDSQEDKDTKET---------EDKDETVPEPVPE
                                              * * : *.  .***...*            : . .   *:* **

T. parva|TP01_0380          DPKDKDTKGPETPQPQPEDPKDKDTK-GPEPMDTEPPKEPEDKDS------------KG
T. annulata|TA20980         EPKDIDHVDVDEPEQEE-QE-DSDKKEDETEDKHEPPKEQEDME-----------AGDT
T. lestoquardi|DQ004498     EPKETEPEPEDPKDTEPETE-KHDSQDDEDTKETEGPKDKKDEKEPETVPEPETPISPGI
                            :**: :     :  : :      . *.: .     . * **: :* .

T. parva|TP01_0380          PETPQPQPEKPEDKDTKGPETPQPEDPKDKDTKGPETPQPQPEDPKDKDTKGPEPMDTEP
T. annulata|TA20980         EQEPQPQPEGQE--------ETEPEVSKDTEPKSEPIQDPIPDTKHEDEQ----------
T. lestoquardi|DQ004498     APEPQPQPEEQ------------PEEPKDQDKEEETVPEPVPEEPKDTEP----------
                               ******               **  ** : :      :* *:  :: :

T. parva|TP01_0380          PKEPEDKDSKGPEDKKDDTKPQPEDPKDKDTKGPEDKKDDTKPQPEDPKDKDSKGTEPMD
T. annulata|TA20980         ---------EQEDSDKEKPDAVPEEP---KD------------------TDHK------
T. lestoquardi|DQ004498     ---------EPEEPK--DTEPEPEEPKETEP------------------EDEK------
                                     :  : .  . .  **:*    .                     * *

T. parva|TP01_0380          TEPPKEPEDKDTKEPEDKD-----SKGTEPMDTESPKEPEKPKEPDGKDPDDHPEEGKKP
T. annulata|TA20980         ---QESEEDKDTKEPEEPTQPTEPEPEPQPQQEVQPEEPTKPQPQETKDPDDKQEEEKKP
T. lestoquardi|DQ004498     ---HDSQEDKDTKETEKTTQPTEPEP--QPQPEERPEEPTKSEPPENKDPDDKQEEEKKS
                                .. ******* *.        .   :*      *:** * :  : *****: ** **

T. parva|TP01_0380          EPGEDKSDQKKEDTKVGTPEKPKPEGDDGLPEKPLPIDQSTPYVPKPDKGGLFQLKTPKL
T. annulata|TA20980         DPGEEDRTDLDKDGQMKTPEKMKPE-DIGGPEFPEPVDQSTPNTVKPPIFDKDRIKTPRL
T. lestoquardi|DQ004498     EPGEEDVVDNEKEGKIKTHEKVETD-DDGGPDFTQAVDQSTPKTVKPPISDKELIKTPRL
                            :***:.  : .:: :: * ** : : * * *:    :***** . **    .   :***:*

T. parva|TP01_0380          VFLTDDENEGPEGPEGPDPLKKKRPFLLYDRPGKTPRRPKKRKIKHLYEDDGSDDDEDED
T. annulata|TA20980         VFLTDDDD-----GEGSEPTKRRKPFLIYDRPGKTPRRPKKKKIKQLFEHDDDGDGDDED
T. lestoquardi|DQ004498     VFLTD-DE-----GEGSEPIKRRKPFLIYDMPGKTPRIPKKKQIRQLFEDDNGDCDEDEE
                            ***** ::      ** :* *:::***:** ****** ***::*::*:*.*... .:**:

T. parva|TP01_0380          EDEDEDEEKEGDDDDGSYKTTGK-DKK-----DDDGSGGSGSGSGTTTTSGSGVVETKQS
T. annulata|TA20980         EDEDEESDE----AEAEIHTSGTKDRKDDGGDKDDGSGGSGGAGPTPST-----DENNQA
T. lestoquardi|DQ004498     K----KKKE----AEKKLPITKT-----------------------PST-----PATIPV
                            :    ...:      : .   : .                       :*        .

T. parva|TP01_0380          QGDGSGSASGSGSGSASGSGSGSASGSGSGSASGSGSGSASGSGDKPKPVRRPLGLPGSS
T. annulata|TA20980         GGSGSGEAKHDDNGDSNTGG-SKQSDTGNGDDSKDKDEKQQ--GEDEKEKEK---EKKEV
T. lestoquardi|DQ004498     TTPGT-------------------------PTKVK------------PISK---IPGKP
                               *:                         :                 :       .

T. parva|TP01_0380          QSKLPIKSSGPGRGPLKTPVATGPGKGGIGSTSG--------ITPV---SSGPLGDPFKT
T. annulata|TA20980         EQKLPVTE-TPSTPAISNPVTKTPKTPKKRRRPSKIPIKQTKVKPASRPSTTPGKGPFKK
T. lestoquardi|DQ004498     TKVKPVSK-IPIRPKNEKPLSKIPGKPTMVKPVSKIPIRAKAVKPVSRPAKTPGKEPSKK
                             .  *:..  *     ..*::. * .        .         :.*.   :. *   * *.

T. parva|TP01_0380          PVSMLPRKVGSKIPVPSKPSKKPRSSSTSSSGKGTGASGSTKTSPKSGDESESKKKRSGG
T. annulata|TA20980         PIVTPG---GPSI---VKPVSRPSTSRPSSTTPPTTT--SGRQPPK------------SD
T. lestoquardi|DQ004498     PKVTPG------------------------------------RETPK------------TE
                            *                                      :  **

T. parva|TP01_0380          PTTVTIPRRPPFVVPGGSKDITTRGTPRTSAPVTPKPE----ETPTPGKRRSETGTKGDP
T. annulata|TA20980         DRRSSQPPRPPFVVPWATKEILTKGATKTPQQEQG-TQFSTEDRTREQGKRAGGETRPDT
T. lestoquardi|DQ004498     DQKPTKPQRPPFVVPGPSKESITRGAQKTPVHQEQRMQASTEETKGEQ-----GKTKTDT
                                : * *******  :*:  *:*: :*         :    :              *: *

T. parva|TP01_0380          SRGGDGDTGSRRSARLLTRGRRNPSPSPTRTGKQGRAGRR--------
T. annulata|TA20980         SKGAVGEGRSRRSQRLSARGTQNTSSSATRETRGARSESTSRNRRNSK
T. lestoquardi|DQ004498     SKGVGNE-----------------------------------------
                            *:*  .:
```
