# Supplementary figures and images for: Identification and characterisation of a Theileria annulata proline‐rich microtubule and SH3 domain‐interacting protein (TaMISHIP) that forms a complex with CLASP1, EB1, and CD2AP at the schizont surface
Source: Cell Microbiol. 2018 Apr 3;20(7):e12838. doi: 10.1111/cmi.12838 (PMC6033098; doi:10.1111/cmi.12838)

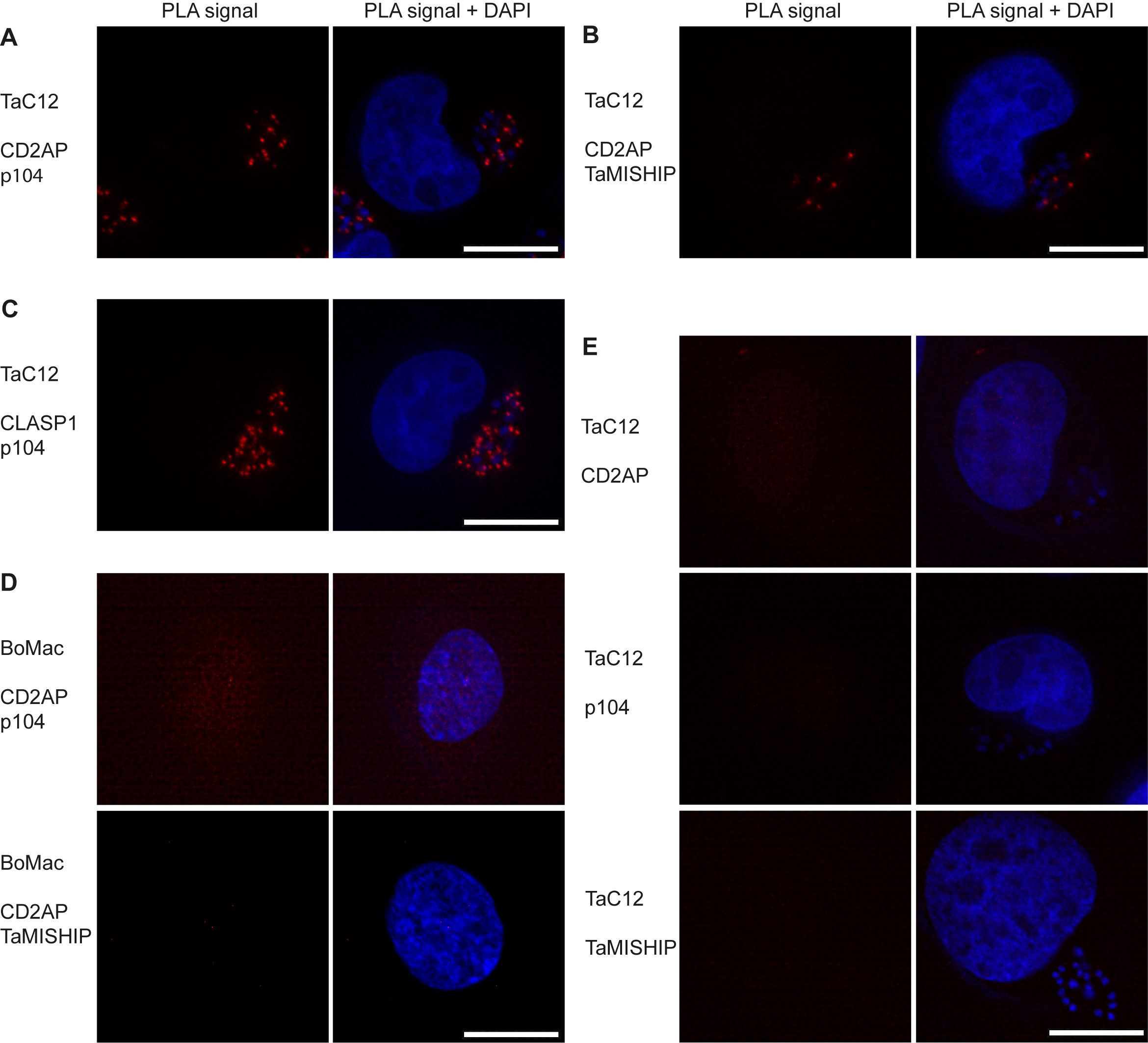

Supplement: Supplementary file 1 — Figure S1. Analysis of myc/BirA*‐CLASP1 1256‐1538 (Western blot) and localization of Nup160, Importin B1, and 14‐3‐3 epsilon in Theileria infected cells. (A) Non‐transduced TaC12 (control) and TaC12_ myc‐BirA*‐CLASP11256–1538 cells were incubated in the presence of 50 μM biotin and subjected to lysis and affinity purification with streptavidin coated beads. The non‐soluble pellet (P), lysate supernatant (SN), pull down flow through (FT) (each 1% of total amount) and 10% of the total streptavidin‐bound protein (pull down, PD) were analyzed with HRP‐conjugated streptavidin. The remaining 90% of streptavidin bound sample was subjected to on‐beads tryptic digest and mass spectrometry analysis. (B) TaC12 cells were transfected with GFP_Nup160 (top panel) or GFP‐Importin B1 (bottom panel). The parasite was labelled with anti‐TaSP (red), and host and parasite nuclei were labelled with DAPI (blue). (C) TaC12 cells were stained with anti‐14‐3‐3 epsilon (green). The parasite was labelled with anti‐p104 (red), and host and parasite nuclei were labelled with DAPI (blue). Scale bar = 10 μm. Figure S2. Analysis of TaC12 cells depleted of CD2AP by shRNA. (A) shRNA targeting CD2AP was expressed in TaC12 cells, and cells were labelled with anti‐CD2AP (green) prior to selection. The parasite was labelled with anti‐p104 (red), host and parasite DNA was labelled with DAPI. One cell is shown which was probably not transduced and still expresses CD2AP, while CD2AP is not detectable in neighboring cells. Scale bar = 10 μm. (B) After selection with puromycin, TaC12 cells expressing a CD2AP targeting shRNA (shRNA), wild type (WT) TaC12 cells, and cells expressing a non‐targeting shRNA (shRNA control) were lysed and analyzed by Western blotting. Anti‐CD2AP antibodies were used to show depletion of CD2AP (runs at around 100 kDa) in the shRNA expressing cell line, the anti‐CD2AP antibody detects unspecific bands at around 80 kDa and 50 kDa. Tubulin was used as a loading control. P is non‐solub [file CMI-20-na-s001.zip › CMI_12838-Sup-00010-FigS10.tif]

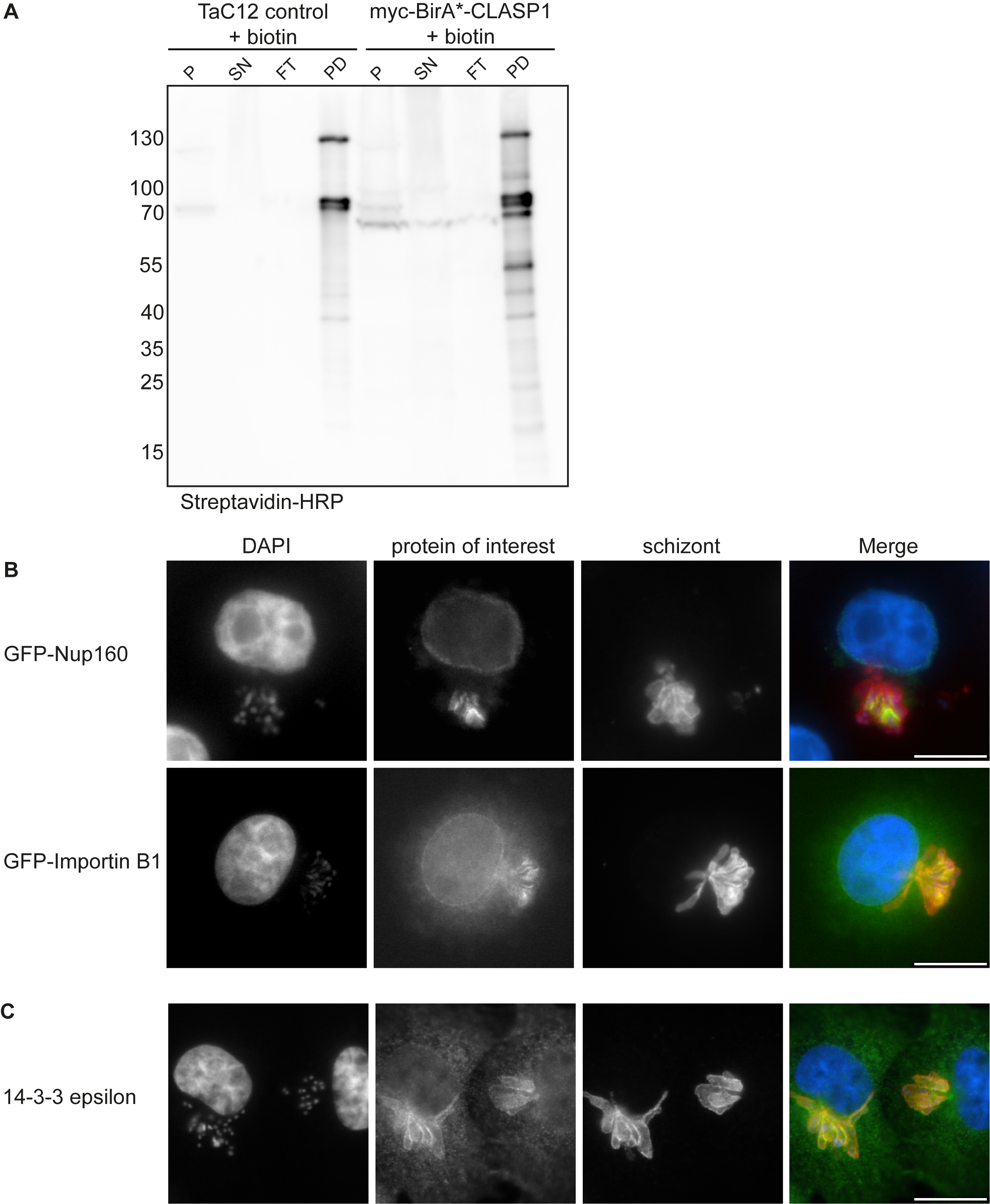

Supplement: Supplementary file 1 — Figure S1. Analysis of myc/BirA*‐CLASP1 1256‐1538 (Western blot) and localization of Nup160, Importin B1, and 14‐3‐3 epsilon in Theileria infected cells. (A) Non‐transduced TaC12 (control) and TaC12_ myc‐BirA*‐CLASP11256–1538 cells were incubated in the presence of 50 μM biotin and subjected to lysis and affinity purification with streptavidin coated beads. The non‐soluble pellet (P), lysate supernatant (SN), pull down flow through (FT) (each 1% of total amount) and 10% of the total streptavidin‐bound protein (pull down, PD) were analyzed with HRP‐conjugated streptavidin. The remaining 90% of streptavidin bound sample was subjected to on‐beads tryptic digest and mass spectrometry analysis. (B) TaC12 cells were transfected with GFP_Nup160 (top panel) or GFP‐Importin B1 (bottom panel). The parasite was labelled with anti‐TaSP (red), and host and parasite nuclei were labelled with DAPI (blue). (C) TaC12 cells were stained with anti‐14‐3‐3 epsilon (green). The parasite was labelled with anti‐p104 (red), and host and parasite nuclei were labelled with DAPI (blue). Scale bar = 10 μm. Figure S2. Analysis of TaC12 cells depleted of CD2AP by shRNA. (A) shRNA targeting CD2AP was expressed in TaC12 cells, and cells were labelled with anti‐CD2AP (green) prior to selection. The parasite was labelled with anti‐p104 (red), host and parasite DNA was labelled with DAPI. One cell is shown which was probably not transduced and still expresses CD2AP, while CD2AP is not detectable in neighboring cells. Scale bar = 10 μm. (B) After selection with puromycin, TaC12 cells expressing a CD2AP targeting shRNA (shRNA), wild type (WT) TaC12 cells, and cells expressing a non‐targeting shRNA (shRNA control) were lysed and analyzed by Western blotting. Anti‐CD2AP antibodies were used to show depletion of CD2AP (runs at around 100 kDa) in the shRNA expressing cell line, the anti‐CD2AP antibody detects unspecific bands at around 80 kDa and 50 kDa. Tubulin was used as a loading control. P is non‐solub [file CMI-20-na-s001.zip › CMI_12838-Sup-0001-FigS1.tif]

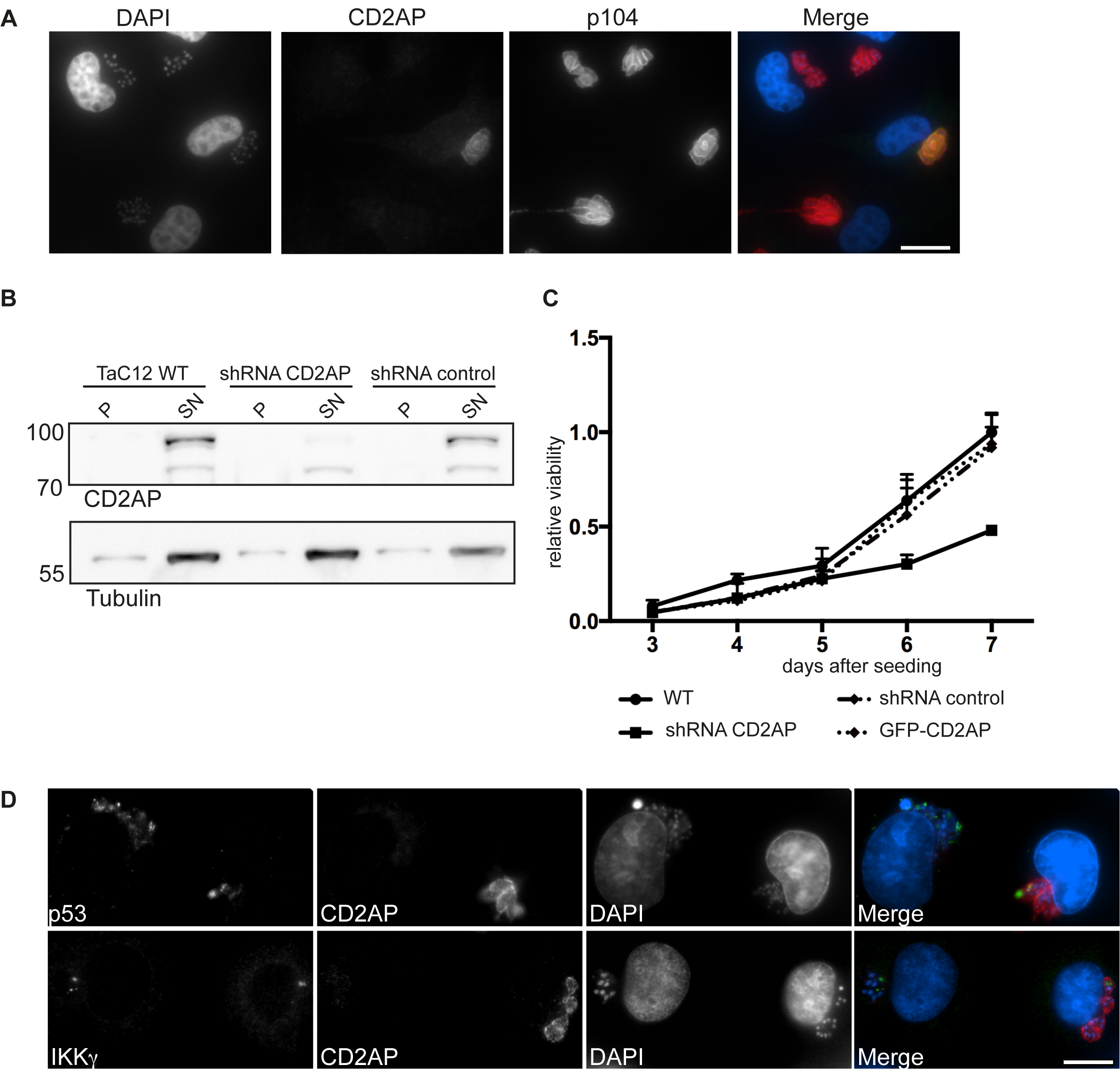

Supplement: Supplementary file 1 — Figure S1. Analysis of myc/BirA*‐CLASP1 1256‐1538 (Western blot) and localization of Nup160, Importin B1, and 14‐3‐3 epsilon in Theileria infected cells. (A) Non‐transduced TaC12 (control) and TaC12_ myc‐BirA*‐CLASP11256–1538 cells were incubated in the presence of 50 μM biotin and subjected to lysis and affinity purification with streptavidin coated beads. The non‐soluble pellet (P), lysate supernatant (SN), pull down flow through (FT) (each 1% of total amount) and 10% of the total streptavidin‐bound protein (pull down, PD) were analyzed with HRP‐conjugated streptavidin. The remaining 90% of streptavidin bound sample was subjected to on‐beads tryptic digest and mass spectrometry analysis. (B) TaC12 cells were transfected with GFP_Nup160 (top panel) or GFP‐Importin B1 (bottom panel). The parasite was labelled with anti‐TaSP (red), and host and parasite nuclei were labelled with DAPI (blue). (C) TaC12 cells were stained with anti‐14‐3‐3 epsilon (green). The parasite was labelled with anti‐p104 (red), and host and parasite nuclei were labelled with DAPI (blue). Scale bar = 10 μm. Figure S2. Analysis of TaC12 cells depleted of CD2AP by shRNA. (A) shRNA targeting CD2AP was expressed in TaC12 cells, and cells were labelled with anti‐CD2AP (green) prior to selection. The parasite was labelled with anti‐p104 (red), host and parasite DNA was labelled with DAPI. One cell is shown which was probably not transduced and still expresses CD2AP, while CD2AP is not detectable in neighboring cells. Scale bar = 10 μm. (B) After selection with puromycin, TaC12 cells expressing a CD2AP targeting shRNA (shRNA), wild type (WT) TaC12 cells, and cells expressing a non‐targeting shRNA (shRNA control) were lysed and analyzed by Western blotting. Anti‐CD2AP antibodies were used to show depletion of CD2AP (runs at around 100 kDa) in the shRNA expressing cell line, the anti‐CD2AP antibody detects unspecific bands at around 80 kDa and 50 kDa. Tubulin was used as a loading control. P is non‐solub [file CMI-20-na-s001.zip › CMI_12838-Sup-0002-FigS2.tif]

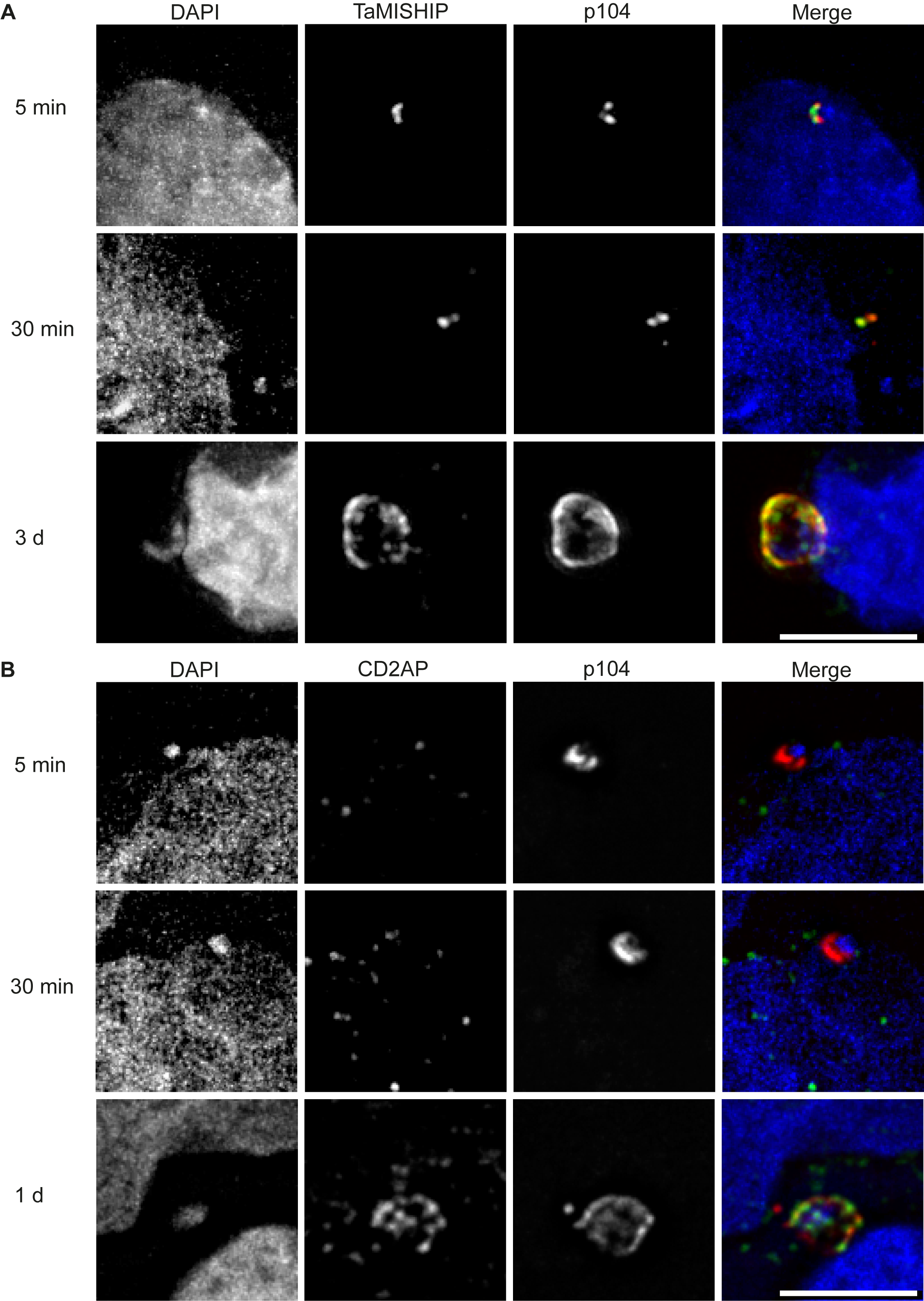

Supplement: Supplementary file 1 — Figure S1. Analysis of myc/BirA*‐CLASP1 1256‐1538 (Western blot) and localization of Nup160, Importin B1, and 14‐3‐3 epsilon in Theileria infected cells. (A) Non‐transduced TaC12 (control) and TaC12_ myc‐BirA*‐CLASP11256–1538 cells were incubated in the presence of 50 μM biotin and subjected to lysis and affinity purification with streptavidin coated beads. The non‐soluble pellet (P), lysate supernatant (SN), pull down flow through (FT) (each 1% of total amount) and 10% of the total streptavidin‐bound protein (pull down, PD) were analyzed with HRP‐conjugated streptavidin. The remaining 90% of streptavidin bound sample was subjected to on‐beads tryptic digest and mass spectrometry analysis. (B) TaC12 cells were transfected with GFP_Nup160 (top panel) or GFP‐Importin B1 (bottom panel). The parasite was labelled with anti‐TaSP (red), and host and parasite nuclei were labelled with DAPI (blue). (C) TaC12 cells were stained with anti‐14‐3‐3 epsilon (green). The parasite was labelled with anti‐p104 (red), and host and parasite nuclei were labelled with DAPI (blue). Scale bar = 10 μm. Figure S2. Analysis of TaC12 cells depleted of CD2AP by shRNA. (A) shRNA targeting CD2AP was expressed in TaC12 cells, and cells were labelled with anti‐CD2AP (green) prior to selection. The parasite was labelled with anti‐p104 (red), host and parasite DNA was labelled with DAPI. One cell is shown which was probably not transduced and still expresses CD2AP, while CD2AP is not detectable in neighboring cells. Scale bar = 10 μm. (B) After selection with puromycin, TaC12 cells expressing a CD2AP targeting shRNA (shRNA), wild type (WT) TaC12 cells, and cells expressing a non‐targeting shRNA (shRNA control) were lysed and analyzed by Western blotting. Anti‐CD2AP antibodies were used to show depletion of CD2AP (runs at around 100 kDa) in the shRNA expressing cell line, the anti‐CD2AP antibody detects unspecific bands at around 80 kDa and 50 kDa. Tubulin was used as a loading control. P is non‐solub [file CMI-20-na-s001.zip › CMI_12838-Sup-0004-FigS4.tif]

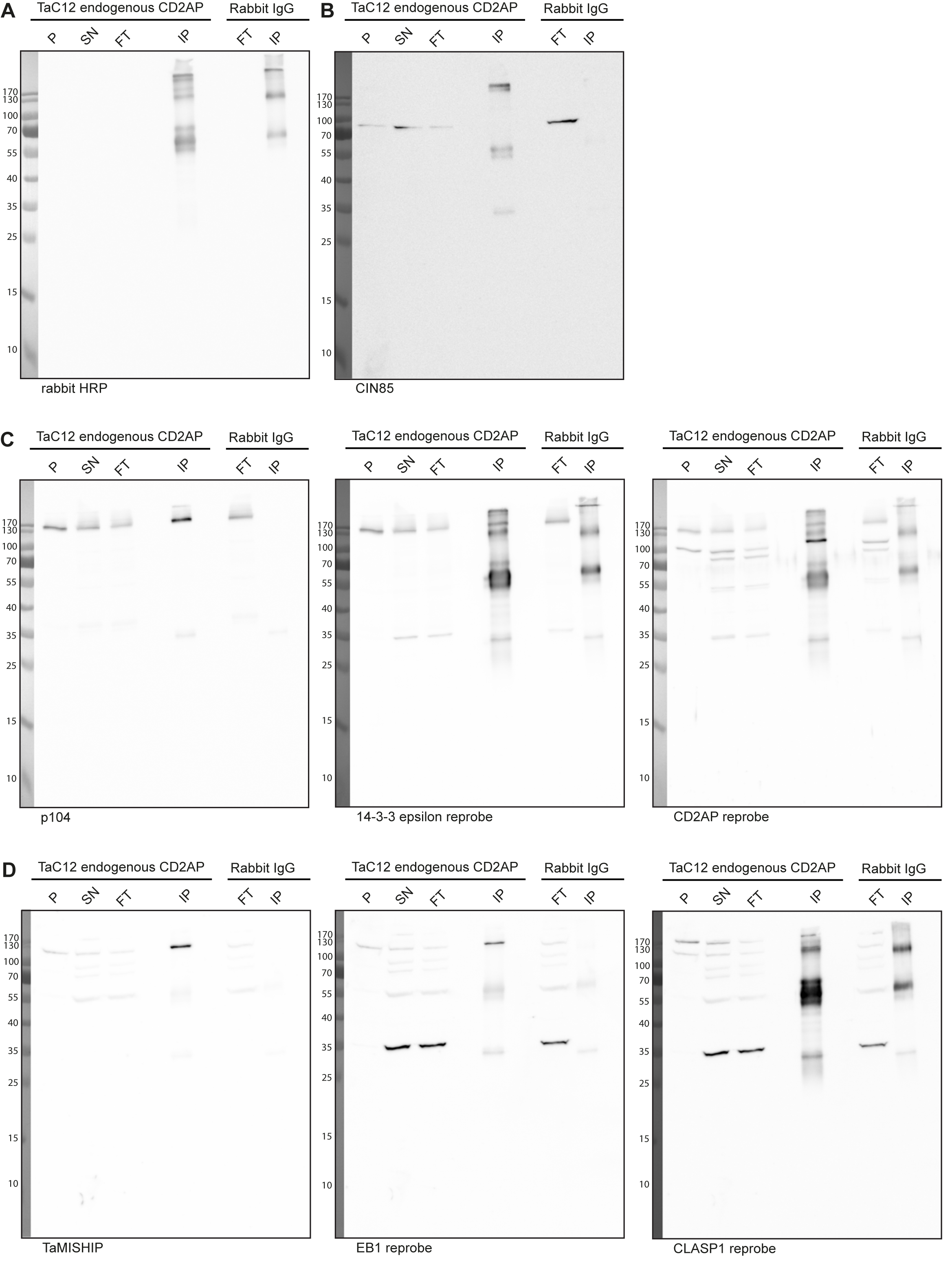

Supplement: Supplementary file 1 — Figure S1. Analysis of myc/BirA*‐CLASP1 1256‐1538 (Western blot) and localization of Nup160, Importin B1, and 14‐3‐3 epsilon in Theileria infected cells. (A) Non‐transduced TaC12 (control) and TaC12_ myc‐BirA*‐CLASP11256–1538 cells were incubated in the presence of 50 μM biotin and subjected to lysis and affinity purification with streptavidin coated beads. The non‐soluble pellet (P), lysate supernatant (SN), pull down flow through (FT) (each 1% of total amount) and 10% of the total streptavidin‐bound protein (pull down, PD) were analyzed with HRP‐conjugated streptavidin. The remaining 90% of streptavidin bound sample was subjected to on‐beads tryptic digest and mass spectrometry analysis. (B) TaC12 cells were transfected with GFP_Nup160 (top panel) or GFP‐Importin B1 (bottom panel). The parasite was labelled with anti‐TaSP (red), and host and parasite nuclei were labelled with DAPI (blue). (C) TaC12 cells were stained with anti‐14‐3‐3 epsilon (green). The parasite was labelled with anti‐p104 (red), and host and parasite nuclei were labelled with DAPI (blue). Scale bar = 10 μm. Figure S2. Analysis of TaC12 cells depleted of CD2AP by shRNA. (A) shRNA targeting CD2AP was expressed in TaC12 cells, and cells were labelled with anti‐CD2AP (green) prior to selection. The parasite was labelled with anti‐p104 (red), host and parasite DNA was labelled with DAPI. One cell is shown which was probably not transduced and still expresses CD2AP, while CD2AP is not detectable in neighboring cells. Scale bar = 10 μm. (B) After selection with puromycin, TaC12 cells expressing a CD2AP targeting shRNA (shRNA), wild type (WT) TaC12 cells, and cells expressing a non‐targeting shRNA (shRNA control) were lysed and analyzed by Western blotting. Anti‐CD2AP antibodies were used to show depletion of CD2AP (runs at around 100 kDa) in the shRNA expressing cell line, the anti‐CD2AP antibody detects unspecific bands at around 80 kDa and 50 kDa. Tubulin was used as a loading control. P is non‐solub [file CMI-20-na-s001.zip › CMI_12838-Sup-0005-FigS5.tif]

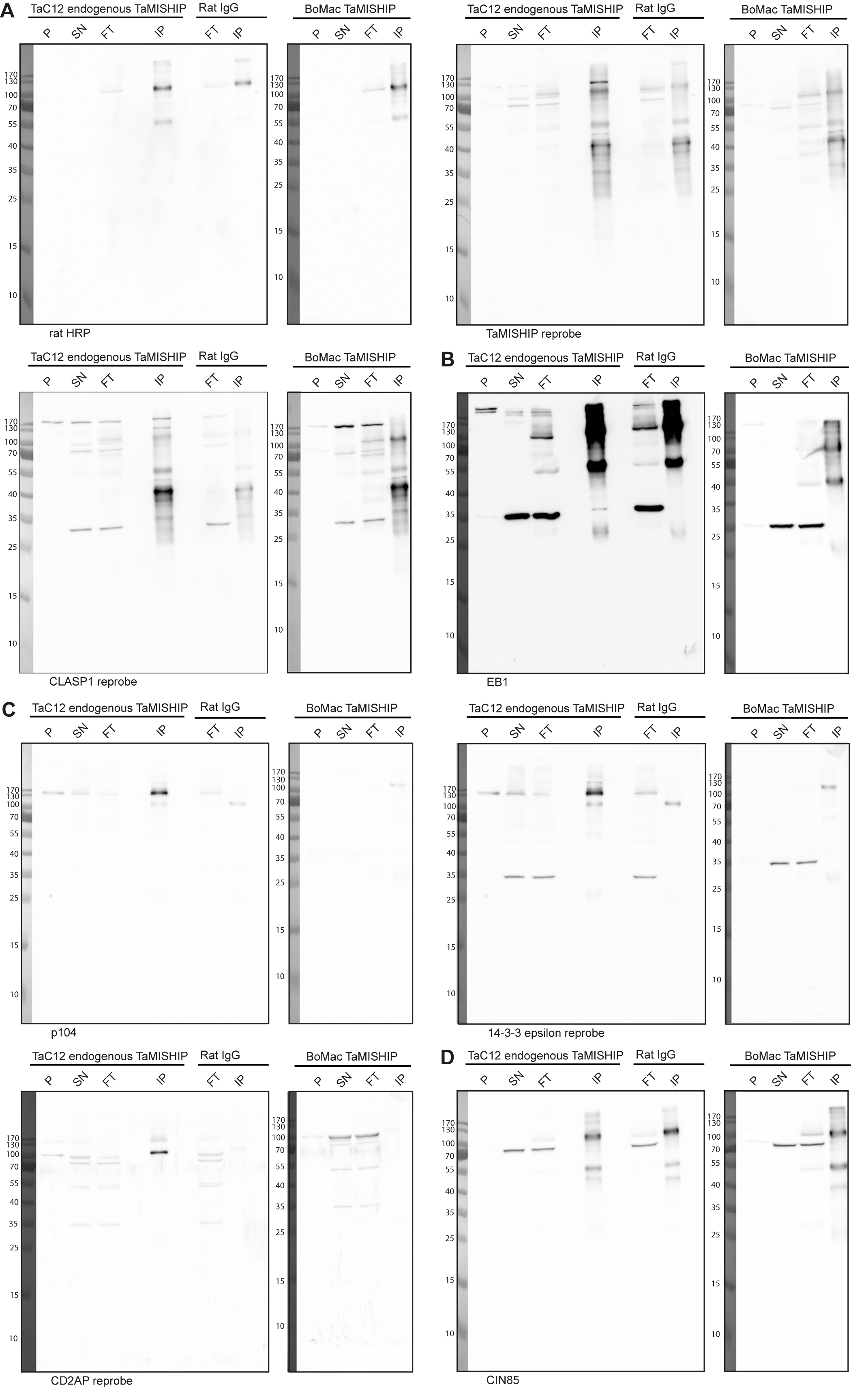

Supplement: Supplementary file 1 — Figure S1. Analysis of myc/BirA*‐CLASP1 1256‐1538 (Western blot) and localization of Nup160, Importin B1, and 14‐3‐3 epsilon in Theileria infected cells. (A) Non‐transduced TaC12 (control) and TaC12_ myc‐BirA*‐CLASP11256–1538 cells were incubated in the presence of 50 μM biotin and subjected to lysis and affinity purification with streptavidin coated beads. The non‐soluble pellet (P), lysate supernatant (SN), pull down flow through (FT) (each 1% of total amount) and 10% of the total streptavidin‐bound protein (pull down, PD) were analyzed with HRP‐conjugated streptavidin. The remaining 90% of streptavidin bound sample was subjected to on‐beads tryptic digest and mass spectrometry analysis. (B) TaC12 cells were transfected with GFP_Nup160 (top panel) or GFP‐Importin B1 (bottom panel). The parasite was labelled with anti‐TaSP (red), and host and parasite nuclei were labelled with DAPI (blue). (C) TaC12 cells were stained with anti‐14‐3‐3 epsilon (green). The parasite was labelled with anti‐p104 (red), and host and parasite nuclei were labelled with DAPI (blue). Scale bar = 10 μm. Figure S2. Analysis of TaC12 cells depleted of CD2AP by shRNA. (A) shRNA targeting CD2AP was expressed in TaC12 cells, and cells were labelled with anti‐CD2AP (green) prior to selection. The parasite was labelled with anti‐p104 (red), host and parasite DNA was labelled with DAPI. One cell is shown which was probably not transduced and still expresses CD2AP, while CD2AP is not detectable in neighboring cells. Scale bar = 10 μm. (B) After selection with puromycin, TaC12 cells expressing a CD2AP targeting shRNA (shRNA), wild type (WT) TaC12 cells, and cells expressing a non‐targeting shRNA (shRNA control) were lysed and analyzed by Western blotting. Anti‐CD2AP antibodies were used to show depletion of CD2AP (runs at around 100 kDa) in the shRNA expressing cell line, the anti‐CD2AP antibody detects unspecific bands at around 80 kDa and 50 kDa. Tubulin was used as a loading control. P is non‐solub [file CMI-20-na-s001.zip › CMI_12838-Sup-0006-FigS6.tif]

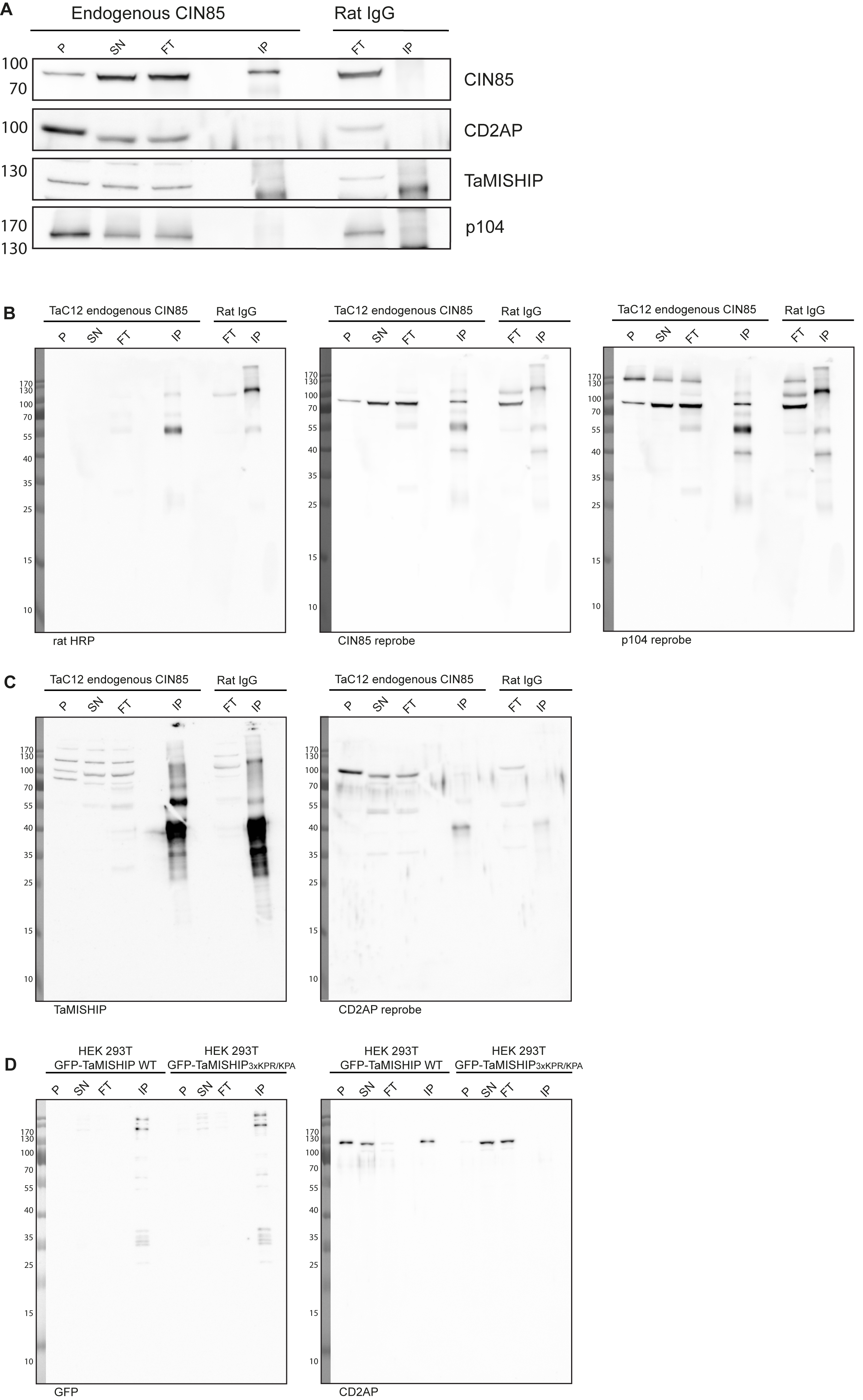

Supplement: Supplementary file 1 — Figure S1. Analysis of myc/BirA*‐CLASP1 1256‐1538 (Western blot) and localization of Nup160, Importin B1, and 14‐3‐3 epsilon in Theileria infected cells. (A) Non‐transduced TaC12 (control) and TaC12_ myc‐BirA*‐CLASP11256–1538 cells were incubated in the presence of 50 μM biotin and subjected to lysis and affinity purification with streptavidin coated beads. The non‐soluble pellet (P), lysate supernatant (SN), pull down flow through (FT) (each 1% of total amount) and 10% of the total streptavidin‐bound protein (pull down, PD) were analyzed with HRP‐conjugated streptavidin. The remaining 90% of streptavidin bound sample was subjected to on‐beads tryptic digest and mass spectrometry analysis. (B) TaC12 cells were transfected with GFP_Nup160 (top panel) or GFP‐Importin B1 (bottom panel). The parasite was labelled with anti‐TaSP (red), and host and parasite nuclei were labelled with DAPI (blue). (C) TaC12 cells were stained with anti‐14‐3‐3 epsilon (green). The parasite was labelled with anti‐p104 (red), and host and parasite nuclei were labelled with DAPI (blue). Scale bar = 10 μm. Figure S2. Analysis of TaC12 cells depleted of CD2AP by shRNA. (A) shRNA targeting CD2AP was expressed in TaC12 cells, and cells were labelled with anti‐CD2AP (green) prior to selection. The parasite was labelled with anti‐p104 (red), host and parasite DNA was labelled with DAPI. One cell is shown which was probably not transduced and still expresses CD2AP, while CD2AP is not detectable in neighboring cells. Scale bar = 10 μm. (B) After selection with puromycin, TaC12 cells expressing a CD2AP targeting shRNA (shRNA), wild type (WT) TaC12 cells, and cells expressing a non‐targeting shRNA (shRNA control) were lysed and analyzed by Western blotting. Anti‐CD2AP antibodies were used to show depletion of CD2AP (runs at around 100 kDa) in the shRNA expressing cell line, the anti‐CD2AP antibody detects unspecific bands at around 80 kDa and 50 kDa. Tubulin was used as a loading control. P is non‐solub [file CMI-20-na-s001.zip › CMI_12838-Sup-0007-FigS7.tif]

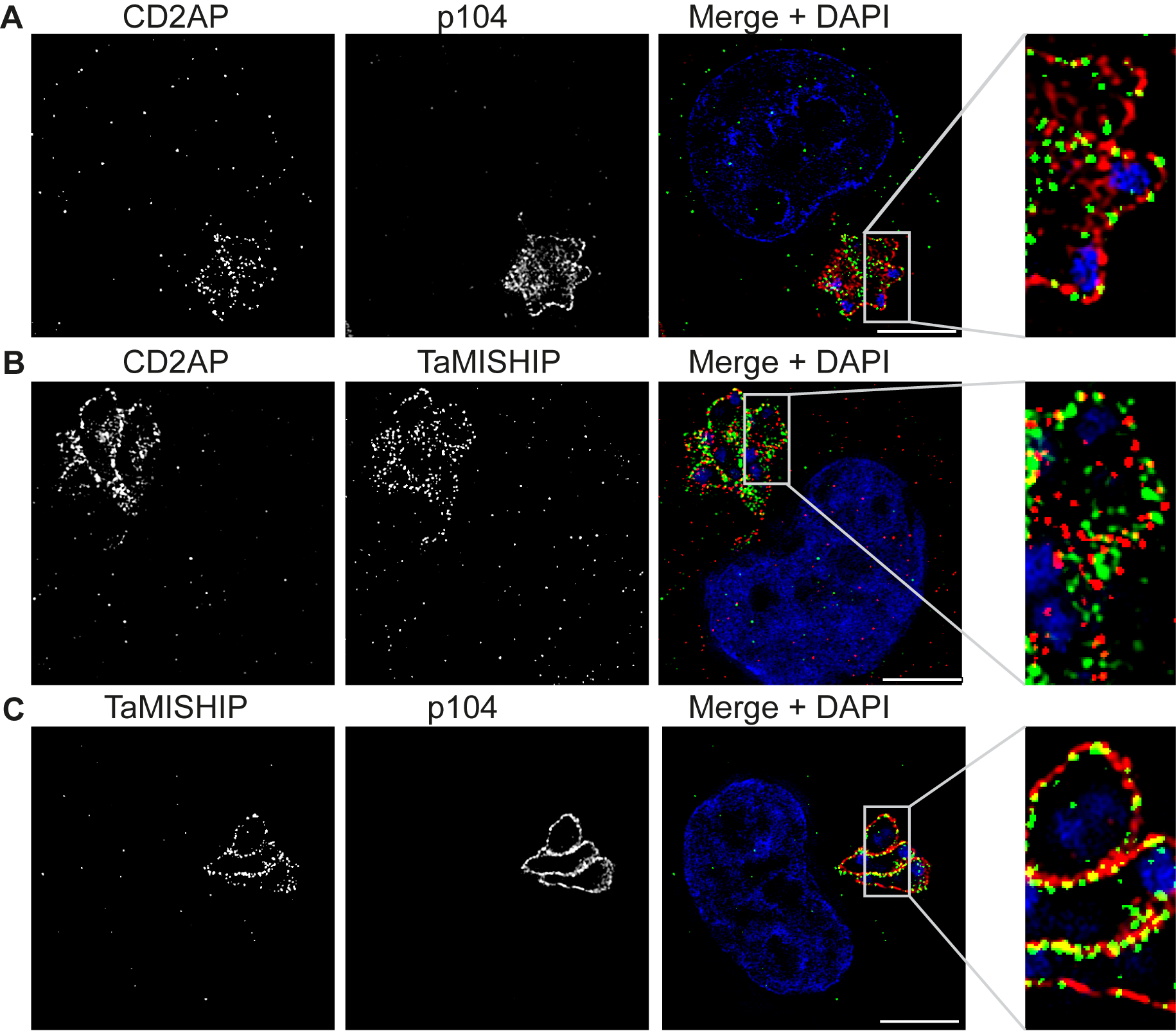

Supplement: Supplementary file 1 — Figure S1. Analysis of myc/BirA*‐CLASP1 1256‐1538 (Western blot) and localization of Nup160, Importin B1, and 14‐3‐3 epsilon in Theileria infected cells. (A) Non‐transduced TaC12 (control) and TaC12_ myc‐BirA*‐CLASP11256–1538 cells were incubated in the presence of 50 μM biotin and subjected to lysis and affinity purification with streptavidin coated beads. The non‐soluble pellet (P), lysate supernatant (SN), pull down flow through (FT) (each 1% of total amount) and 10% of the total streptavidin‐bound protein (pull down, PD) were analyzed with HRP‐conjugated streptavidin. The remaining 90% of streptavidin bound sample was subjected to on‐beads tryptic digest and mass spectrometry analysis. (B) TaC12 cells were transfected with GFP_Nup160 (top panel) or GFP‐Importin B1 (bottom panel). The parasite was labelled with anti‐TaSP (red), and host and parasite nuclei were labelled with DAPI (blue). (C) TaC12 cells were stained with anti‐14‐3‐3 epsilon (green). The parasite was labelled with anti‐p104 (red), and host and parasite nuclei were labelled with DAPI (blue). Scale bar = 10 μm. Figure S2. Analysis of TaC12 cells depleted of CD2AP by shRNA. (A) shRNA targeting CD2AP was expressed in TaC12 cells, and cells were labelled with anti‐CD2AP (green) prior to selection. The parasite was labelled with anti‐p104 (red), host and parasite DNA was labelled with DAPI. One cell is shown which was probably not transduced and still expresses CD2AP, while CD2AP is not detectable in neighboring cells. Scale bar = 10 μm. (B) After selection with puromycin, TaC12 cells expressing a CD2AP targeting shRNA (shRNA), wild type (WT) TaC12 cells, and cells expressing a non‐targeting shRNA (shRNA control) were lysed and analyzed by Western blotting. Anti‐CD2AP antibodies were used to show depletion of CD2AP (runs at around 100 kDa) in the shRNA expressing cell line, the anti‐CD2AP antibody detects unspecific bands at around 80 kDa and 50 kDa. Tubulin was used as a loading control. P is non‐solub [file CMI-20-na-s001.zip › CMI_12838-Sup-0009-FigS9.tif]
